# Supplementary material for: Allosteric regulation and crystallographic fragment screening of SARS-CoV-2 NSP15 endoribonuclease
Source: Nucleic Acids Res. 2023 Apr 28;51(10):5255–70. doi: 10.1093/nar/gkad314 (PMC10250223; doi:10.1093/nar/gkad314)
Supplement: gkad314_Supplemental_Files [file gkad314_supplemental_files.zip › Supplementary datasets.zip!/183/Refine_4/01-BUSTER/Cycle-2/LIST.html]

Refining

**BUSTER 2.X**: A suite of programs for macromolecular structure refinement by maximum-likelihood and Bayesian methods  
Copyright © 1992-2020 Global Phasing Ltd.  
All rights reserved.  
Authors: G. Bricogne, E. Blanc, M. Brandl, C. Flensburg, P. Keller, W. Paciorek, P. Roversi, O. Smart, C. Vonrhein, T. Womack  

Please reference use of the software as:

- BUSTER-TNT 2.X, Global Phasing Ltd, Sheraton House, Cambridge CB3 0AX, UK

Version: 2.10.3 for linux64-ifort made on May 20 2020 at 01:14:34  
Licenced to: Diamond Light Source, Scientific Software  

```
---


### Parsing of the BUSTER cardfile

TITLE : autoBUSTER refinement

   Cell information

   Symmetry information

 Using CCP4 X-ray form factors.

   Missing structure chemical composition

 Using ATOM block for chemical composition.


   Shell information

### Parsing of the TNT cardfile


---


### Reading observations file

 Number of reflexions in input:  70521

      Number of accepted reflexions:  64732

      Number of rejected reflexions:      0

   Reflexions in test set:            3243

   Reflexions in working set:        61489


---


### 1. Generating initial model

 2.10 * Shannon sampling rate. NX NY NZ:   288  288  240


  Effective Shannon rates are:    2.15    2.15    2.45


   Allocated BUFFER of size:     76.465 Mb.

   Initial Geometry Information


 No missing atoms declared


   Summary of model volumes

 No missing atoms declared: no prior computed


 Computing bulk solvent model scattering


 NCSMASK PDB radius for solvent mask:     2.15 Angstrom

 running PDBSET (setting cell and spacegroup P1) ... done
 running NCSMASK [1] (creating mask, SMOOTH 1.04563) ... done
 running MAPDUMP (to check if reduction to unit cell is needed) ... done
 running MAPMASK (converting mask to map) ... done

   Generation of babslv by theta-filter

   Electron density in frg mask:  0.420 Å-1

 FOBS  put on rough absolute scale by Wilson plot :
           K        =  0.51687E+02
           B        =     -6.205

 

### Cycle 0001: MaxLik scaling and refinement


   MaxLik Scaling Cycle 0001

   Wilson plot

   Statistics in resolution bins just after scaling
```

|  |  |
| --- | --- |
|  | - Rxpct factors plot- Rxpct factors table- <Log-Lik Gain>- Ln Sigma A- Correlation Coefficients |

```
   <Log-Likelihood> of null-hypothesis

Structure Factor Amplitudes


   Average Figure of Merit

 Switching off refinement of overall temperature factor


 Added B =    -0.3409 Å2 to all atoms in fragment


 Added B =    -0.3409 Å2 to B_SOLV

```
gelly refinement module
 Authors (2004-2009) O. S. Smart, C. Flensburg, W. Paciorek, C. Vonrhein, 
                     M. Brandl, T. O. Womack and G. Bricogne
         (2010-2011) O. S. Smart, C. Flensburg, W. Paciorek, C. Vonrhein, 
                     T. O. Womack and G. Bricogne
         (2012-2014) O. S. Smart, C. Flensburg, W. Paciorek, C. Vonrhein, 
                     and G. Bricogne
         (2015-2020) G. Bricogne, C. Flensburg, W. Paciorek, C. Vonrhein
 Copyright © 2004-2020 Global Phasing Ltd. 
 All rights reserved.
 gelly module compilation date May 20 2020 01:11:20
 
 gelly uses routines from TNT geometry. References: 
     Tronrud, D. E., Ten Eyck, L. F., Matthews, B. W. (1987)
     "An Efficient General-Purpose Least-Squares Refinement Program 
     for Macromolecular Structures". Acta Crystallogr A, 43:489-501.
     Tronrud, D. E. (1997). "The TNT Refinement Package", 
     in Macromolecular Crystallography, Part B, Eds Charlie Carter 
     and Robert Sweet, Methods in Enzymology, 277:306-319.
 wildcard matching function by Julian Robichaux http://www.nsftools.com

 No -random_seed flag found. Random number stream initialised using:
2147483562
2147483398

 Control flags set to:  -autoncs -hydrogens_input_occ2zero \
                     -hydrogens_output_occ2heavy -screen \
                     100 -screen_sigma \
                     3.0 -keeppdb \
                     1000000 -taomonitor_verbose \
                     2 
 

 ############### Starting setup via TNT routines

 Setting up tnt routines including license check:

                   TNT Stereochemistry Program
 Version: 5.22.3.0
 Licence for Diamond Light Source, Scientific Software is valid
 
 Using TNT geometry routines to load the tnt control file: gelly.tnt
 Using TNT routines to load initial coordinates from file fragment.cor
 Expecting 6125 atoms

 Found     2202 "NOTE BUSTER_RESIDUE_ATOM_DATA" cards
 Have copied information for    6125 atoms from TNT to gelly's store 
 
 The TNT WEIGHT cards used by this run:
 -----WEIGHT       BOND     2.0
 -----WEIGHT       ANGLE    2.0
 -----WEIGHT       IMPROPER 0.0
 -----WEIGHT       TORSION  2.0
 -----WEIGHT       PSEUDO   0.0
 -----WEIGHT       TRIGONAL 2.0
 -----WEIGHT       PLANE    5.0
 -----WEIGHT       CONTACT  5.0
 -----WEIGHT       BCORREL  10.0
 -----WEIGHT       CHIRAL   5.0
 -----WEIGHT       NCS      50.0
 -----WEIGHT       IDEAL    4.0
 -----WEIGHT       RFACTOR  2.18
 
 Dictionary versions picked up from NOTE BUSTER_RESTRAINT_DICT cards as: 
 ------ protgeo_eh99.dat (V1.6.4.17) 20191001  STANDARD AMINO ACID DICTIONARY. BONDS AND ANGLES FROM ENGH AND HUBER EH99. OTHER VALUES BASED ON PREVIOUS TNT OR TAKEN FROM CCP4. INCLUDES HYDROGEN ATOMS.
 ------ exoticaa.dat (V1.3.4.15) 20200327  COLLECTION OF NON-STANDARD AMINO ACIDS, MAINLY EH91 WITHOUT IDEAL DISTANCE INFO
 ------ nuclgeo.dat (V1.13.4.8) 20190909
 ------ bcorrel.dat (V1.15.10.2) 20190616
 ------ contact.dat (V1.15.12.10) 20191121
 ------ idealdist_contact.dat (V1.3.4.12) 20191001  IDEAL-DISTANCE CONTACT TERM DATA AS USED IN PROLSQ. VALUES USED HERE ARE BASED ON DATA HARVESTING OF PDB AS OF 20190109.
 ------ restraints for LIG (Unknown) from cif dictionary Z2856434903.cif
 ------ restraints for CIT (CITRIC ACID) from cif dictionary CIT.cif; generated by GRADE_PDB_LIGAND 1.2.12 (October 4 2016) using MOGUL 1.7.2(RC1), CSD as538be, with quantum mechanics RM1 ((DatabaseID PDB-CIT))
 ------ assume.dat (V1.9.4.2) 20180705
 
 Have picked up spacegroup name for pdb output as 'P 63      '
 Setting up Chain/Residue arrays
 
 Have command line specification -autoncs*
     Have found       1 NCS relations
     Pairwise matching residue number and type between chains: 
                    A        B        S    
          A         .       99%       -   
          B                  .        -   
          S                           .   
       Table key--                                                       
          all   : two chains have 100% match and same number of residues 
         multi  : ditto and they contribute to a multi chain ncs cluster 
          ***%  : two chains have >80% matching residue number/type      
           -    : chains appear to be unrelated                          
      chain A     have identified ncs to     1 other chains. So will weight each autoncs by (       6.666/#)=      6.6660
      chain B     have identified ncs to     1 other chains. So will weight each autoncs by (       6.666/#)=      6.6660
      chain S     have identified ncs to     0 other chains. 
     For .Gelly cards produced see below - after pruning
 
 Setting up BUSTER_SET groups
 Have found    89 BUSTER_SET cards
 Now silently applying automatic/dictionary set definitions. For a verbose listing use '-verbose_set' flag.
 Applying user defined set definitions:
 =====NOTE BUSTER_SET ncsautoXcld = Water --> set 'ncsautoXcld' now has   581 atoms 
 =====NOTE BUSTER_SET ncsautoABset = Chain_B \ ncsautoXcld --> set 'ncsautoABset' now has  2774 atoms 
 Have defined    48 sets: 
      set:                 'All' has   6125 atoms in   1281 residues - automatically defined
      set:               'Empty' has      0 atoms in      0 residues - automatically defined
      set:            'ExoticAA' has      0 atoms in      0 residues - defined by dictionary file
      set:           'ExoticNUC' has      0 atoms in      0 residues - defined by dictionary file
      set:              'NotHET' has   5514 atoms in    698 residues - defined by dictionary file
      set:             'Chain_A' has   2770 atoms in    350 residues - automatically defined
      set:             'Chain_B' has   2774 atoms in    350 residues - automatically defined
      set:             'Chain_S' has    581 atoms in    581 residues - automatically defined
      set:               'Water' has    581 atoms in    581 residues - defined by dictionary file
      set:          'StdProtein' has   5514 atoms in    698 residues - defined by dictionary file
      set:             'Protein' has   5514 atoms in    698 residues - defined by dictionary file
      set:                'Back' has   2794 atoms in    698 residues - defined by dictionary file
      set:                'Side' has   2720 atoms in    650 residues - defined by dictionary file
      set:        'RnaCanonical' has      0 atoms in      0 residues - defined by dictionary file
      set:        'DnaCanonical' has      0 atoms in      0 residues - defined by dictionary file
      set:     'RnaDnaCanonical' has      0 atoms in      0 residues - defined by dictionary file
      set:     'DnaRnaCanonical' has      0 atoms in      0 residues - defined by dictionary file
      set:         'RnaExtended' has      0 atoms in      0 residues - defined by dictionary file
      set:         'DnaExtended' has      0 atoms in      0 residues - defined by dictionary file
      set:      'RnaDnaExtended' has      0 atoms in      0 residues - defined by dictionary file
      set:      'DnaRnaExtended' has      0 atoms in      0 residues - defined by dictionary file
      set:                 'Dna' has      0 atoms in      0 residues - defined by dictionary file
      set:                 'Rna' has      0 atoms in      0 residues - defined by dictionary file
      set:              'RnaDna' has      0 atoms in      0 residues - defined by dictionary file
      set:              'DnaRna' has      0 atoms in      0 residues - defined by dictionary file
      set:             'Nucleic' has      0 atoms in      0 residues - defined by dictionary file
      set:             'Polymer' has   5514 atoms in    698 residues - defined by dictionary file
      set: 'BufferWeichenberger' has     13 atoms in      1 residues - defined by dictionary file
      set:    'BufferAdditional' has      0 atoms in      0 residues - defined by dictionary file
      set:              'Buffer' has     13 atoms in      1 residues - defined by dictionary file
      set:         'MetalAlkali' has      0 atoms in      0 residues - defined by dictionary file
      set:  'MetalAlkalineEarth' has      0 atoms in      0 residues - defined by dictionary file
      set:     'MetalTransition' has      0 atoms in      0 residues - defined by dictionary file
      set: 'MetalPostTransition' has      0 atoms in      0 residues - defined by dictionary file
      set:    'MetalLanthanides' has      0 atoms in      0 residues - defined by dictionary file
      set:               'Metal' has      0 atoms in      0 residues - defined by dictionary file
      set:         'SugarAlphaD' has      0 atoms in      0 residues - defined by dictionary file
      set:         'SugarAlphaL' has      0 atoms in      0 residues - defined by dictionary file
      set:               'Sugar' has      0 atoms in      0 residues - defined by dictionary file
      set:               'Other' has     30 atoms in      2 residues - defined by dictionary file
      set:            'Hydrogen' has      0 atoms in      0 residues - defined by dictionary file
      set:         'nonHydrogen' has   6125 atoms in   1281 residues - defined by dictionary file
      set:             'OccZero' has      0 atoms in      0 residues - defined by dictionary file
      set:          'OccNonZero' has   6125 atoms in   1281 residues - defined by dictionary file
      set:             'OccFull' has   6076 atoms in   1278 residues - defined by dictionary file
      set:          'OccNonFull' has     49 atoms in      3 residues - defined by dictionary file
      set:         'ncsautoXcld' has    581 atoms in    581 residues - user specified
      set:        'ncsautoABset' has   2774 atoms in    350 residues - user specified
 No command line arguments '-occ_allhet' or '-occ_restyp' so will not setup automated occcupancy refinement
 
 Interpreting CONSTANT and FREE cards: 
 =====CONSTANT     OCC
 Have found and interpreted      1 CONSTANT cards in the TNT input. 
 Before any COMBINE - Number of atoms with fixed XYZ =     0
 Before any COMBINE - Number of atoms with fixed B   =     0
 Before any COMBINE - Number of atoms with fixed OCC =  6125
 Before any COMBINE - Number of atoms with fixed BIJ =   581
                            cf total number of atoms =  6125
 
 There are no COMBINE cards to be found in the TNT card input 
 
 There are no NOTE BUSTER_COMBINE_RES cards to be found in the TNT card input 

 ############### Working out full connectivity list


 ############### Working out equivalent atoms


 Residue with most atoms given below:
        B|401:C01(LIG)      
        B|401:N02(LIG)      
        B|401:C03(LIG)      
        B|401:C04(LIG)      
        B|401:N05(LIG)      
        B|401:C06(LIG)      
        B|401:C07(LIG)      
        B|401:C08(LIG)      
        B|401:C09(LIG)      
        B|401:C10(LIG)      
        B|401:C11(LIG)      
        B|401:N12(LIG)      
        B|401:C13(LIG)      
        B|401:O14(LIG)      
        B|401:C15(LIG)      
        B|401:C16(LIG)      
        B|401:C17(LIG)      


 Have no '-equiv_check_plus' argument - so will take atom type into
 account when checking for equivalence of otherwise identical atoms.
 

 ############### Looking at dihedrals


 ############### Looking at chirals

 NOTE BUSTER_CHIRAL_ANGLECUT card found - read value=    -31.00 degs from it
 NOTE BUSTER_CHIRAL_SIGMA card found - read value=      1.70 degs from it
 RANGE allowed for B variables in refinement:   from      3.00000 to    300.00000

 ############### Preparing for PDB output


 ############### Processing extra restraints explicitly defined

 No 'NOTE BUSTER_TARGET' cards or -target?? args found (used to read coordinates files for soft ncs to external)
 No 'NOTE BUSTER_OCCSUM'     cards found - so not using sum of occupancy restraints
 No 'NOTE BUSTER_DISTANCE'   cards found - so not setting up any additional atom-atom distance restraints
 No 'NOTE BUSTER_UTILANGLE'  cards found - so not setting up any additional restraints on any angle
 No 'NOTE BUSTER_UTILTOR'    cards found - so not setting up any additional sinusoidal restraints on torsions
 
 --- Have some similarity definitions 
     Similarity restraints on RMSD use superposition routines from: 
     Coutsias, E.A., Seok, C., Dill, K.A.(2004)
     "Using quaternions to calculate RMSD", J. Comput. Chem., 25:1849-1857 

 Have found    1 'NOTE BUSTER_SIM_DEFINE' cards 
     Maximum number of chains involved in any SIM group (excluding the template)=     1


 ########## Interpreting card: NOTE BUSTER_SIM_DEFINE ncsautoAB ncsautoABset A
     Number of atoms in template set=  2774
     Number of related chains (other than template) in SIM set=    1
     List of chains to be restrained (excluding template)=   A   
     Pairwise RMSD, XYZ in Angstroms, for SIM group: ncsautoAB
                 B         A   
     B         -----     ----- 
     A         0.696     ----- 
     Overall RMSD,XYZ for the SIM group is     0.696 Angstroms
     Pairwise rms deviation of [individual B's minus their set/chain average] in Angs**2
                 B         A         Bavg
     B         -----     -----     63.74
     A         22.28     -----     77.64
     Overall RMSD,B   for the SIM group is    22.281 Angs**2
     The number of common atoms for the above fits:
                 B         A   
     B         -----     ----- 
     A          2757     ----- 
     (compared to the template set that has    2774 atoms in it)
     List of atoms that are absent in one or more chains for this group: 
                                    B    A   
                    C01     401     +    -
                    N02     401     +    -
                    C03     401     +    -
                    C04     401     +    -
                    N05     401     +    -
                    C06     401     +    -
                    C07     401     +    -
                    C08     401     +    -
                    C09     401     +    -
                    C10     401     +    -
                    C11     401     +    -
                    N12     401     +    -
                    C13     401     +    -
                    O14     401     +    -
                    C15     401     +    -
                    C16     401     +    -
                    C17     401     +    -
     (key  +  atom present for this chain 
           -  atom absent  for this chain)
 transforms for each of the superpositions orthogonal matrix r11 r12 r13 r21 r22 r23 r31 r32 r33 tx ty tz (PDBSET syntax): 
TRANS_BA -0.877829 -0.478847  0.011043 -0.478791  0.877897  0.007460 -0.013267  0.001261 -0.999911    45.358   -12.820    15.701
 reverse transforms 
TRANS_AB -0.877829 -0.478791 -0.013267 -0.478847  0.877897  0.001261  0.011043  0.007460 -0.999911    33.887    32.955    15.295
 
 Setting up LSSR interatomic distance restraints
 Interpreting card: NOTE BUSTER_SIM_RESTRAIN_LSSR ncsautoAB 6.666
        setting up interatomic distance pair restraints with:
        Dmin=         0.00
        Dmax=         5.50
        sigma=        0.20
        Vmax=         3.00
        weight=       6.67
        number of distance pairs defined for this set is      35316
 Have not supplied either '-sim_swap_equiv' or '-sim_swap_equiv_plus' argument 
   so will not try swaping equivalent atoms to improve NCS and/or target agreement.
 
 NCS by LSSR outlier analysis
 -autoncs option automatic NCS prune routine -------- 
 Residues pruned out by gradient condition:
           A|54     A|253      B|54     B|253
  
 Residues pruned out by function/max condition:
          A|149     A|150     A|173     A|187     A|188     A|215     A|220 
          A|234     A|244     A|259     A|264     A|345    A|ENDA     B|149 
          B|150     B|173     B|187     B|188     B|215     B|220     B|234 
          B|244     B|259     B|264     B|345    B|ENDA 
  
 Total number of residues for which LSSR restraints have been pruned out is       30 out of      698 participating
 Number of LSSR restraints pruned out is      2182 this is    6.2% of the total           35316
  
 -autoncs .Gelly cards suitable for adaptation for manual definition/pruning
           (note auto pruning is done in a slightly different way so exact results may differ slightly)
 -autoncs option .Gelly cards also written to file  auto_gelly_cards.txt
NOTE BUSTER_SET ncsautoXcld = Water    # card added - produced by -autoncs
NOTE BUSTER_SET ncsautoXcld = ncsautoXcld + { B|54 B|149 B|150 B|173 B|187 }    # card added - produced by -autoncs
NOTE BUSTER_SET ncsautoXcld = ncsautoXcld + { B|188 B|215 B|220 B|234 B|244 }    # card added - produced by -autoncs
NOTE BUSTER_SET ncsautoXcld = ncsautoXcld + { B|253 B|259 B|264 B|345 B|ENDA }    # card added - produced by -autoncs
NOTE BUSTER_SET ncsautoABset = Chain_B \ ncsautoXcld    # card added - produced by -autoncs
NOTE BUSTER_SIM_DEFINE ncsautoAB ncsautoABset A    # card added - produced by -autoncs
NOTE BUSTER_SIM_RESTRAIN_LSSR ncsautoAB 6.666    # card added - produced by -autoncs

 LSSR function contribution before pruning    137380.0
                             after pruning    116391.6
 Graph of which residues pruned from LSSR: ncs_pruned.mtv
 Graph of LSSR function outliers ncs_function_outliers.mtv
 Graph of LSSR gradient outliers ncs_gradient_outliers.mtv
 LSSR - analysis of distance difference distribution for NCS
         DeltaD less than 0.5 Angs:  proportion of population= 96.28% average DeltaD of subset     0.104 Angs
 Histogram of LSSR deltaD distribution ncs_histogram.mtv
 
 Interpreting card: NOTE BUSTER_TLS_SET tls1 { A|* }
 Interpreting card: NOTE BUSTER_TLS_SET tls2 { B|* }
 Interpreting card: NOTE BUSTER_TLS_SET tls1 { A|* }
 Interpreting card: NOTE BUSTER_TLS_T   tls1     -1.40902E-01 -6.30068E-02  2.40854E-01 -1.40248E-02  2.99908E-02 -4.86377E-03
 Interpreting card: NOTE BUSTER_TLS_L   tls1      4.94961E-01  2.74622E-01  7.99820E-02  2.76942E-01 -2.11335E-01  5.62449E-04
 Interpreting card: NOTE BUSTER_TLS_S   tls1      2.26571E-01 -1.51776E-01  8.73531E-02 -1.03155E-01 -2.74707E-02 -1.84204E-02 -5.17057E-02  1.25425E-02
 Interpreting card: NOTE BUSTER_TLS_O   tls1     -2.33748E+01 -8.36624E+01  1.14308E+01
 Interpreting card: NOTE BUSTER_TLS_SET tls2 { B|* }
 Interpreting card: NOTE BUSTER_TLS_T   tls2     -1.42296E-01 -1.01915E-01  3.03964E-01  1.65150E-02  4.29271E-02  1.39210E-02
 Interpreting card: NOTE BUSTER_TLS_L   tls2      5.71646E-01  2.05044E-01  3.60703E-01  3.24162E-01 -3.15261E-01 -1.73112E-01
 Interpreting card: NOTE BUSTER_TLS_S   tls2     -1.36019E-02  5.36055E-02 -3.99431E-02  1.31338E-02  1.12817E-01 -1.79603E-02 -2.03873E-02  1.93526E-02
 Interpreting card: NOTE BUSTER_TLS_O   tls2      2.20570E+01 -7.19562E+01  3.49479E+00
 Interpreting card: NOTE BUSTER_TLS_FIX tls1 ALL
 Interpreting card: NOTE BUSTER_TLS_FIX tls2 ALL


 TLS information. Total number of groups:     2


 Group   Natoms    FixRB   FixTLS   Group Tag    Group Spec
     1     2770      Y        Y     tls1    { A|* }
     2     2774      Y        Y     tls2    { B|* }
 
NOTE BUSTER_TLS_SET tls1 { A|* }
NOTE BUSTER_TLS_T   tls1     -1.40902E-01 -6.30068E-02  2.40854E-01 -1.40248E-02  2.99908E-02 -4.86377E-03
NOTE BUSTER_TLS_L   tls1      4.94961E-01  2.74622E-01  7.99820E-02  2.76942E-01 -2.11335E-01  5.62449E-04
NOTE BUSTER_TLS_S   tls1      2.26571E-01 -1.51776E-01  8.73531E-02 -1.03155E-01 -2.74707E-02 -1.84204E-02 -5.17057E-02  1.25425E-02
NOTE BUSTER_TLS_O   tls1     -2.33748E+01 -8.36624E+01  1.14308E+01
NOTE BUSTER_TLS_FIX tls1 ALL
 
NOTE BUSTER_TLS_SET tls2 { B|* }
NOTE BUSTER_TLS_T   tls2     -1.42296E-01 -1.01915E-01  3.03964E-01  1.65150E-02  4.29271E-02  1.39210E-02
NOTE BUSTER_TLS_L   tls2      5.71646E-01  2.05044E-01  3.60703E-01  3.24162E-01 -3.15261E-01 -1.73112E-01
NOTE BUSTER_TLS_S   tls2     -1.36019E-02  5.36055E-02 -3.99431E-02  1.31338E-02  1.12817E-01 -1.79603E-02 -2.03873E-02  1.93526E-02
NOTE BUSTER_TLS_O   tls2      2.20570E+01 -7.19562E+01  3.49479E+00
NOTE BUSTER_TLS_FIX tls2 ALL


 TLS: mmCIF group specification begin


#
loop_
_pdbx_refine_tls_group.id
_pdbx_refine_tls_group.refine_tls_id
_pdbx_refine_tls_group.beg_PDB_ins_code
_pdbx_refine_tls_group.beg_auth_asym_id
_pdbx_refine_tls_group.beg_auth_seq_id
_pdbx_refine_tls_group.end_PDB_ins_code
_pdbx_refine_tls_group.end_auth_asym_id
_pdbx_refine_tls_group.end_auth_seq_id
_pdbx_refine_tls_group.selection
_pdbx_refine_tls_group.selection_details
   1   1 . A   -1 . A  346 . '{ A|* }'
   2   1 . A  401 . A  401 . '{ A|* }'
   3   2 . B   -1 . B  346 . '{ B|* }'
   4   2 . B  401 . B  401 . '{ B|* }'
#


 TLS: mmCIF group specification end


 Interpreting card: NOTE BUSTER_ANISO_SET Empty
 Interpreting card: NOTE BUSTER_ANISO_WEIGHT SCA  16.0
 Interpreting card: NOTE BUSTER_ANISO_WEIGHT SPH   0.6    0.6
 Interpreting card: NOTE BUSTER_ANISO_WEIGHT SIM   7.8    4.8    2.6    0
 Interpreting card: NOTE BUSTER_ANISO_WEIGHT RIG  21.5   21.5    3.7    0


 After TLS and ANISO: Number of atoms with fixed/combined xyz =     0
 After TLS and ANISO: Number of atoms with fixed/combined B   =     0
 After TLS and ANISO: Number of atoms with fixed/combined OCC =  6125
 After TLS and ANISO: Number of atoms with fixed/combined BIJ =  6125
 After TLS and ANISO: Number of atoms in a TLS group          =  5544
                                     cf total number of atoms =  6125
 

 ############### Setup state vector

 No free_vect_ulim_xyz argument found: using default of  100.000
 No free_vect_llim_xyz argument found: using default of -100.000
 Number of parameters added for TLS description                 =       0
 Number of parameters to describe molecular conformation        =   24500

 ############### Setup free vector

 Number of free parameters to be adjusted in refinement   =   24500

 ############### Setting up own geometry


 No command line argument '-tnt_fn' so will use gelly to do geometry function calculations

 ============= Call TNT functions


 ============= Get TNT symmetry information

 
      Getting symmetry operators from TNT. 
          gelly will classify symmetry using pdb-like convention: 
               SYMOP   SYMMETRY
              NNNMMM   OPERATOR
                1555   X,Y,Z
                2555   -Y,X-Y,Z
                3555   -X+Y,-X,Z
                4555   -X,-Y,1/2+Z
                5555   Y,-X+Y,1/2+Z
                6555   X-Y,X,1/2+Z
          where NNN -> operator number and MMM -> translation vector
 

 ============= Own Gelly geometry/restraints setup


 ----------- Identify bond-types


    Setting up GEOMBONDREC_TYPE_HASH* to be able to map a given bond type (e.g.
    "single") to an internal integer (GBOND_TYPE=1) etc.

    Using default  GEOMBONDREC_TYPE_HASH_STR='SING* 1 DOUB* 2 TRIP* 3 AROM* 4 DELO* 4 METAL* 5':
    to override default use a NOTE BUSTER_GEOMBONDREC_TYPE_HASH_STR gelly card.

 ----------- Classify torsion into peptide omega and other


 ----------- Setup of non-bonded terms

 Ideal-distance contact term setup:
    Restraint function constants (normally these values are from NOTE BUSTER_IDEAL_CONTACT_* cards)
    Sigma for general contacts      0.200 Angs
    Sigma for 1-4 contacts          0.100 Angs (contacts across a torsion)
    Sigma for 1-5 contacts          0.150 Angs
    Sigma for 1-N contacts          0.200 Angs
    Sigma for HBOND pairs           0.100 Angs (contacts between HB_types A and D or B's)
    Contact distance increment for each atom in HBOND          -0.150 Angs 
    Contact distance increment for H-bond acceptor              0.100 Angs 
    Contact distance increment for 1-4 with an O atom          -0.050 Angs 
    Contact distance increment for 1-4 with an N atom          -0.180 Angs 
    Contact distance increment for 1-4 with an C atom          -0.370 Angs 
    Contact distance increment for 1-4 with an S atom          -0.150 Angs 
    Contact distance increment for 1-4 with an P atom          -0.150 Angs 
    Contact distance increment for 1-4 with any other atom     -0.150 Angs 
    All atoms matched for CCP4 atom type.
    Number of CCP4 atom types used is         24 (H-bound and not H-bound counted separately)

 Setting up short/ideal-distance contacts
   Using gelly special position handling. (For TNT instead rerun with argument '-special_tnt')

   No command line argument '-special_all' specified. We will auto-generate
   EXCLUDE cards only for atom-atom contacts across symmetry (i.e. on special
   position) when this involves a contact within a residue to itself.

   Have set up non-bonded exclusions - 1-3 contacts
   Atom A|27:CA         makes the most 1-3 contacts:  6

   Have set up non-bonded exclusions - 1-4 contacts
   Atom A|27:N          makes the most 1-4 contacts:  7

   Have set up non-bonded exclusions - 1-5 contacts
   Atom A|28:C.A        makes the most 1-5 contacts: 10

 ############################################################################################################
 ###################################################### setting up contact terms
 ############################################################################################################


 ============= Call Gelly versions of TNT functions

 WARNING: 1-4 contact without 1-(2,3)-4 data for A|401:O2(CIT)        and A|401:C3(CIT)        X-X
 WARNING: 1-4 contact without 1-(2,3)-4 data for A|401:C2(CIT)        and A|401:O6(CIT)        X-X
 WARNING: 1-4 contact without 1-(2,3)-4 data for B|341:C(PHE)         and B|342:CB(TYR)        X-X
 WARNING: 1-4 contact without 1-(2,3)-4 data for B|401:C01(LIG)       and B|401:C06(LIG)       X-X
 WARNING: 1-4 contact without 1-(2,3)-4 data for B|401:C03(LIG)       and B|401:C06(LIG)       X-X
 WARNING: 1-4 contact without 1-(2,3)-4 data for B|401:C04(LIG)       and B|401:C07(LIG)       X-X
 WARNING: 1-4 contact without 1-(2,3)-4 data for B|401:C04(LIG)       and B|401:C17(LIG)       X-X
 WARNING: 1-4 contact without 1-(2,3)-4 data for B|401:C06(LIG)       and B|401:C17(LIG)       X-X

       TERM      weight      number      TNT_fn    Gelly_fn        diff
       BOND       2.000        5660    5869.083    5869.083       0.000
       ANGL       2.000        7681   13328.570   13328.570       0.000
       IMPR       0.000           0       0.000       0.000       0.000
       TORS       2.000        1968    3374.204    3374.204      -0.000
       TRIG       2.000           0       0.000       0.000       0.000
       PLAN       5.000         964   15822.564   15822.564       0.000
       CONT       5.000           0     493.868       0.000     493.868
       BCOR      10.000        5630  147658.939  150177.439   -2518.500


 ============= Call Gelly geometry functions

   Switching torsion functional form to sinusoidal rather than split harmonic functional.
      if you want this not to happen then rerun with '-torsharm'
      Weight for sinusoidal torsion=     2.000
   BCOR function/gradient will be split between H and non-H atoms (as for any atom pair). Use '-bcorrel_all_in_hydrogens' to change this.

 ----------- Fix B-correlation restraints

 
 Checking for bonds that do not have a bcorrel restraint defined
 We will wipe all existing BCORrels if OCC=0.0 atom(s) is/are involved. This can be' switched off via the '-bcorrel_nowipe_zeroocc' flag.
 We will wipe all existing BCORrels if only non-hydrogen atom(s) with fixed B(iso) are involved. This can be switched off via the '-bcorrel_nowipe_fixb_nonhydrogens' flag.
 We will keep all existing BCORrels if hydrogen atom(s) with fixed B(iso) is/are involved. This can be switched off via the '-bcorrel_wipe_fixb_hydrogens' flag.
 No -bcorrel_missing_sigma found. So will set sigma to     2.5 Ang**2 for any bond with no existing BCORrel
 No -bcorrel_missing_sigma_hydrogens found. So will set sigma to     1.0 Ang**2 for any bond with no existing BCORrel involving hydrogens

 No active BCORels found involving OCC=0.0 atom(s)


 No active BCORels found involving fixed-B (only) non-hydrogen atom(s)

    add a default BCOR for bond A|401:C1(CIT) A|401:C2(CIT)
    add a default BCOR for bond A|401:C1(CIT) A|401:O1(CIT)
    add a default BCOR for bond A|401:C1(CIT) A|401:O2(CIT)
    add a default BCOR for bond A|401:C2(CIT) A|401:C3(CIT)
    add a default BCOR for bond A|401:C3(CIT) A|401:C4(CIT)
    add a default BCOR for bond A|401:C3(CIT) A|401:C6(CIT)
    add a default BCOR for bond A|401:C3(CIT) A|401:O7(CIT)
    add a default BCOR for bond A|401:C4(CIT) A|401:C5(CIT)
    add a default BCOR for bond A|401:C5(CIT) A|401:O3(CIT)
    add a default BCOR for bond A|401:C5(CIT) A|401:O4(CIT)
    add a default BCOR for bond A|401:C6(CIT) A|401:O5(CIT)
    add a default BCOR for bond A|401:C6(CIT) A|401:O6(CIT)
    add a default BCOR for bond B|401:C01(LIG) B|401:N02(LIG)
    add a default BCOR for bond B|401:N02(LIG) B|401:C03(LIG)
    add a default BCOR for bond B|401:N02(LIG) B|401:C07(LIG)
    add a default BCOR for bond B|401:C03(LIG) B|401:C04(LIG)
    add a default BCOR for bond B|401:C04(LIG) B|401:N05(LIG)
    add a default BCOR for bond B|401:N05(LIG) B|401:C06(LIG)
    add a default BCOR for bond B|401:N05(LIG) B|401:C08(LIG)
    add a default BCOR for bond B|401:C06(LIG) B|401:C07(LIG)
    add a default BCOR for bond B|401:C08(LIG) B|401:C09(LIG)
    add a default BCOR for bond B|401:C08(LIG) B|401:C17(LIG)
    add a default BCOR for bond B|401:C09(LIG) B|401:C10(LIG)
    add a default BCOR for bond B|401:C10(LIG) B|401:C11(LIG)
    add a default BCOR for bond B|401:C11(LIG) B|401:N12(LIG)
    add a default BCOR for bond B|401:C11(LIG) B|401:C16(LIG)
    add a default BCOR for bond B|401:N12(LIG) B|401:C13(LIG)
    add a default BCOR for bond B|401:C13(LIG) B|401:O14(LIG)
    add a default BCOR for bond B|401:C13(LIG) B|401:C15(LIG)
    add a default BCOR for bond B|401:C16(LIG) B|401:C17(LIG)

 Have added a total of        30 BCORrels for bonds which do not already have a BCORrel


 No bonds (for potential BCORrel addition) were skipped due to both atoms having constant/fixed B(iso)


 ----------- Deactivate hydrogen-free planes if hydrogen atoms present

 A total of       0 planes have been deactivated because they are duplicated hydrogen-free and with-hydrogen forms.

 ----------- Handle peptide omega angle(s)

      Torsion angles split into peptide omega and other
         Number of peptide omega angles is       698
         Number of other torsion is             1270
      Peptide omega angles are monitored rather than directly restrained (PLANE used instead).

 ----------- Fix cis-PRO

 Now apply the fix to get correct restraints for cis PRO residues
   Have got      11 bonds records and      26 angles
   from GEOMETRY cards for the special PROC residue
   Weight for ideal-distance contact term=     4.000

 ----------- Prepare SSBOND/LINK for output PDB file


 ----------- Prepare for QM


 ----------- Prepare symmetry in utility distance restraints


 ############### Setting up Rhofit contacts (if needed)

 No ligand-(fixed protein) contact term will be used

 ############### Working out initial function and gradient


   MaxLik Scaling Cycle 0002
 
 Initial functional value = 5.081158E+05 rms gradient=      724.537
 X-ray contribution to function value = 1.723797E+05
 Geometry contribution (inc ncs)      = 3.357362E+05
 Geometry function summary
     term                           number     weight       rms        GooF      function     fn/numb
     ================================================================================================
     BOND bond lengths (angs)       5660       2.000      0.0144       0.720      5869.08       1.037
     ANGL bond angles (degs)        7681       2.000        1.56       0.931     13328.57       1.735
     TORS split-harmonic tors       1270       0.000       18.32       1.153         0.00       0.000
     SINTOR sinusoidal tors         1270       2.000       18.32                  2597.30       2.045
     PLAN general plane (angs)       964       5.000      0.0362       1.812     15822.56      16.413
     BCORrelations (angs^2)         5660      10.000       4.627       1.652    154403.33      27.280
     CONT (bad contacts)               0       5.000                                 0.00
     IDEAL(ideal-dist contact)      5186       4.000                             27276.86
     CHIRAL (gelly semiharmon)       740       5.000                                46.87
     SIM similarity restraint for NCS and/or TARGET                             116391.62
     OCCSUM restraint on sum of occupanies                                           0.00       as none defined
     DISTAN utility restraints interatomic distance                                  0.00       as none defined
     UTILANGLE utility angle restraints                                              0.00       as none defined
     UTILTOR utility restraints on torsion angles                                    0.00       as none defined
     Peptide omega torsion angles                           7.80 degs - monitor only
 
 WARNING: Have |delta/sigma| deviations > 5.0 sigma. Number of outliers for each term: 
 WARNING:         1 bond lengths. Worst is     8.3 sigs     1.50 Angs B|342:C(TYR)=B|343:N(PRO)
 WARNING:         9 bond angles.  Worst is     8.2 sigs   131.65 degs B|342:C(TYR)=B|343:N(PRO)=B|343:CA(PRO)
 WARNING:        17 planes.       Worst is     7.5 sigs     0.15 Angs B|190:C=B|190:CA=B|190:O=B|191:N=B|191:CA
 WARNING:         8 idealD contacts. Worst     6.7 sigs     2.74 Angs B|297:C(LEU)=B|298:C(LEU)
 WARNING:        76 Bcorrel.      Worst is    13.2 sigs   -33.10 Angs^2 A|401:C6=O5 (CIT)

 Weighted rms      0.0144         1.564  (temporary rms bond and angle for BUSTER to pick up!)
 Weighted intra-residual rms(bond) for StdProtein (excluding H)      0.0147
 Weighted intra-residual rms(bond) for Buffer     (excluding H)      0.0273

 ############### Running initial check


 Initial Geometry Sanity Check

 Check for duplicate bonds: no duplicates found
 
 Check for duplicate or missing bond angles: all are OK
 Check for bond between an atom and itself not involving symmetry:  all are OK
 *** WARNING: There are 1 bad contacts where Delta/sigma is greater than      6.5
 *** WARNING: Looking at each one in turn to see whether it is likely to indicate a setup problem with geometry restraints.

        1 dist=   2.74 idealD=   3.03 sigma=   0.04 abs(Delta/sigma)=    6.67
          Between atoms   B|297:C (LEU)           and   B|298:C (LEU)           (C-C)
          This is a contact between two 'Protein' type atoms from distinct
          residues.
 [check-042]
          Not between atoms that normally join residues in proteins. So
          probably just a bad contact (maybe due to a poor or very distorted
          input model).
 GEOMETRY CHECK OVERALL:  WARN


 ############### Creating geometry/screen report


 ############### Option "tao" - run Tao optimizer

 TAO: Toolkit for Advanced Optimization. Reference: 
     Munson, T., Sarich, J., Wild, S., Benson, S., McInnes, L.C. (2018)
     "TAO User Manual (Revision 3.9)", Mathematics and Computer Science
     Division, Argonne National Laboratory
     ANL/MCS-TM-322, http://www.mcs.anl.gov/petsc/
 
 Have found -keeppdb flag so will write out a .pdb file every 1000000 its during opt
 Will apply TAO optimization 
 Maximum number of iterations      100
 Convergence test on Grms       4.0000 (-glim)
 Limit on rms displacement to original position (rmsD2init): NONE test inactive (-dlim)

    analysing argument no 0 : -autoncs with j=0
    analysing argument no 1 : -hydrogens_input_occ2zero with j=0
    analysing argument no 2 : -hydrogens_output_occ2heavy with j=0
    analysing argument no 3 : -screen with j=0
    analysing argument no 4 : 100 with j=0
    analysing argument no 5 : -screen_sigma with j=0
    analysing argument no 6 : 3.0 with j=0
    analysing argument no 7 : -keeppdb with j=0
    analysing argument no 8 : 1000000 with j=0
    analysing argument no 9 : -taomonitor_verbose with j=0
    found argument starting with "-tao" : -taomonitor_verbose
    analysing argument no 10 : 2 with j=1
    found value after -tao/-options_/-viewer_ argument : "2"
 PETSC/TAO: setting option "-taomonitor_verbose" = "2"


   MaxLik Scaling Cycle 0003
   Iters     Tot_Funct    Xray_Funct    Geom_Funct          Grms     rmsD2init     maxD2init             R         Rfree           LLG       LLGfree     rms(bond)    rms(angle)       Opttag_H
   Iters             K   B_IMPF_FRAG        K_MISS   B_TEMP_MISS   K_IMPF_MISS   B_IMPF_MISS        K_SOLV   B_TEMP_SOLV   K_IMPF_SOLV   B_IMPF_SOLV        B_TEMP     B_TEMP_11     B_TEMP_12     B_TEMP_22     B_TEMP_13     B_TEMP_23     B_TEMP_33      K_WILSON      B_WILSON      FGCYCLES       Scatag_H
   Iters          BOND         ANGLE      IMPROPER       TORSION    SINTORSION      TRIGONAL        PLANAR  BCORRELATION       CONTACT     IDEALDIST        CHIRAL    SIMILARITY       OCCSSUM  UTILDISTANCE     UTILANGLE   UTILTORSION        RHOFIT        ADP-01        ADP-02        ADP-03        ADP-04        ADP-05        ADP-06        ADP-07        ADP-08        ADP-09        ADP-10      RHOFITPC            QM       Funtag_H
       0  5.081158E+05  1.723797E+05  3.357362E+05       724.537  0.000000E+00  0.000000E+00      0.201795      0.209849  0.000000E+00  0.000000E+00     0.0144486       1.56356       Opttag_D
       0       58.6998       2.56065       1.00000  0.000000E+00       1.00000  0.000000E+00      0.879496       66.2153       1.00000       18.7159  0.000000E+00       3.84543  0.000000E+00       3.84543  0.000000E+00  0.000000E+00      -7.69087       57.8987       1.83292        2       Scatag_D
       0       5869.08       13328.6  0.000000E+00  0.000000E+00       2597.30  0.000000E+00       15822.6  1.544033E+05  0.000000E+00       27276.9       46.8719  1.163916E+05 -1.000000E+10 -1.000000E+10 -1.000000E+10 -1.000000E+10 -1.000000E+10  0.000000E+00  0.000000E+00  0.000000E+00  0.000000E+00  0.000000E+00  0.000000E+00  0.000000E+00  0.000000E+00  0.000000E+00  0.000000E+00  0.000000E+00  0.000000E+00       Funtag_D
   Geometry Restraint Deviations SCREEN Information

 

   MaxLik Scaling Cycle 0004

   MaxLik Scaling Cycle 0005

   Wilson plot

   Statistics in resolution bins after refinement cycle 0001
```


|  |  |
| --- | --- |
|  | - Rxpct factors plot- Rxpct factors table- <Log-Lik Gain>- Ln Sigma A- Correlation Coefficients |


```
   Iters     Tot_Funct    Xray_Funct    Geom_Funct          Grms     rmsD2init     maxD2init             R         Rfree           LLG       LLGfree     rms(bond)    rms(angle)
   Iters             K   B_IMPF_FRAG        K_MISS   B_TEMP_MISS   K_IMPF_MISS   B_IMPF_MISS        K_SOLV   B_TEMP_SOLV   K_IMPF_SOLV   B_IMPF_SOLV        B_TEMP     B_TEMP_11     B_TEMP_12     B_TEMP_22     B_TEMP_13     B_TEMP_23     B_TEMP_33      K_WILSON      B_WILSON      FGCYCLES
   Iters          BOND         ANGLE      IMPROPER       TORSION    SINTORSION      TRIGONAL        PLANAR  BCORRELATION       CONTACT     IDEALDIST        CHIRAL    SIMILARITY       OCCSSUM  UTILDISTANCE     UTILANGLE   UTILTORSION        RHOFIT        ADP-01        ADP-02        ADP-03        ADP-04        ADP-05        ADP-06        ADP-07        ADP-08        ADP-09        ADP-10      RHOFITPC            QM
       1  4.213421E+05  1.729706E+05  2.483715E+05       504.666    0.00736919     0.0831125      0.202936      0.210745   -0.00440818   -0.00342881     0.0113465       1.41855       Opttag_D
       1       58.6071       2.61631       1.00000  0.000000E+00       1.00000  0.000000E+00      0.879969       66.0908       1.00000       18.3996  0.000000E+00       4.14064  0.000000E+00       4.14064  0.000000E+00  0.000000E+00      -8.28127       57.8987       1.83292        2       Scatag_D
       1       3619.42       10970.9  0.000000E+00  0.000000E+00       2586.45  0.000000E+00       13725.9       85133.1  0.000000E+00       25224.5       21.5346  1.070896E+05 -1.000000E+10 -1.000000E+10 -1.000000E+10 -1.000000E+10 -1.000000E+10  0.000000E+00  0.000000E+00  0.000000E+00  0.000000E+00  0.000000E+00  0.000000E+00  0.000000E+00  0.000000E+00  0.000000E+00  0.000000E+00  0.000000E+00  0.000000E+00       Funtag_D
   Geometry Restraint Deviations SCREEN Information

 

   MaxLik Scaling Cycle 0006

   Wilson plot

   Statistics in resolution bins after refinement cycle 0002
```


|  |  |
| --- | --- |
|  | - Rxpct factors plot- Rxpct factors table- <Log-Lik Gain>- Ln Sigma A- Correlation Coefficients |


```
   Iters     Tot_Funct    Xray_Funct    Geom_Funct          Grms     rmsD2init     maxD2init             R         Rfree           LLG       LLGfree     rms(bond)    rms(angle)
   Iters             K   B_IMPF_FRAG        K_MISS   B_TEMP_MISS   K_IMPF_MISS   B_IMPF_MISS        K_SOLV   B_TEMP_SOLV   K_IMPF_SOLV   B_IMPF_SOLV        B_TEMP     B_TEMP_11     B_TEMP_12     B_TEMP_22     B_TEMP_13     B_TEMP_23     B_TEMP_33      K_WILSON      B_WILSON      FGCYCLES
   Iters          BOND         ANGLE      IMPROPER       TORSION    SINTORSION      TRIGONAL        PLANAR  BCORRELATION       CONTACT     IDEALDIST        CHIRAL    SIMILARITY       OCCSSUM  UTILDISTANCE     UTILANGLE   UTILTORSION        RHOFIT        ADP-01        ADP-02        ADP-03        ADP-04        ADP-05        ADP-06        ADP-07        ADP-08        ADP-09        ADP-10      RHOFITPC            QM
       2  3.814838E+05  1.732230E+05  2.082608E+05       354.208     0.0129290     0.0757822      0.203567      0.211192   -0.00629089   -0.00455548    0.00988350       1.32293       Opttag_D
       2       58.5461       2.63604       1.00000  0.000000E+00       1.00000  0.000000E+00      0.880141       65.8686       1.00000       18.3084  0.000000E+00       4.31776  0.000000E+00       4.31776  0.000000E+00  0.000000E+00      -8.63551       57.8987       1.83292        1       Scatag_D
       2       2746.25       9541.83  0.000000E+00  0.000000E+00       2574.84  0.000000E+00       12135.7       58757.5  0.000000E+00       23721.1       20.4812       98763.2 -1.000000E+10 -1.000000E+10 -1.000000E+10 -1.000000E+10 -1.000000E+10  0.000000E+00  0.000000E+00  0.000000E+00  0.000000E+00  0.000000E+00  0.000000E+00  0.000000E+00  0.000000E+00  0.000000E+00  0.000000E+00  0.000000E+00  0.000000E+00       Funtag_D
   Geometry Restraint Deviations SCREEN Information

 
       3  3.479996E+05  1.737449E+05  1.742547E+05       303.883     0.0238267      0.124855      0.204862      0.212165    -0.0101850   -0.00727890     0.0116707       1.26111       Opttag_D
       3       58.4780       2.68685       1.00000  0.000000E+00       1.00000  0.000000E+00      0.880533       65.5073       1.00000       18.2108  0.000000E+00       4.59768  0.000000E+00       4.59768  0.000000E+00  0.000000E+00      -9.19537       57.8987       1.83292        1       Scatag_D
       3       3829.22       8670.80  0.000000E+00  0.000000E+00       2558.43  0.000000E+00       9682.98       44079.6  0.000000E+00       21468.0       61.6152       83904.0 -1.000000E+10 -1.000000E+10 -1.000000E+10 -1.000000E+10 -1.000000E+10  0.000000E+00  0.000000E+00  0.000000E+00  0.000000E+00  0.000000E+00  0.000000E+00  0.000000E+00  0.000000E+00  0.000000E+00  0.000000E+00  0.000000E+00  0.000000E+00       Funtag_D
       4  3.286648E+05  1.741296E+05  1.545351E+05       315.755     0.0329724      0.149100      0.205819      0.212845    -0.0130549   -0.00901943     0.0130902       1.38283       Opttag_D
       4       58.4243       2.72258       1.00000  0.000000E+00       1.00000  0.000000E+00      0.880711       65.1803       1.00000       18.2157  0.000000E+00       4.77011  0.000000E+00       4.77011  0.000000E+00  0.000000E+00      -9.54023       57.8987       1.83292        1       Scatag_D
       4       4817.41       10425.5  0.000000E+00  0.000000E+00       2547.94  0.000000E+00       8132.87       36440.1  0.000000E+00       20055.7       102.416       72013.2 -1.000000E+10 -1.000000E+10 -1.000000E+10 -1.000000E+10 -1.000000E+10  0.000000E+00  0.000000E+00  0.000000E+00  0.000000E+00  0.000000E+00  0.000000E+00  0.000000E+00  0.000000E+00  0.000000E+00  0.000000E+00  0.000000E+00  0.000000E+00       Funtag_D
       5  3.117693E+05  1.743557E+05  1.374136E+05       241.146     0.0403227      0.157707      0.206416      0.213275    -0.0147415   -0.00990791     0.0125545       1.31309       Opttag_D
       5       58.3752       2.73884       1.00000  0.000000E+00       1.00000  0.000000E+00      0.880684       64.8715       1.00000       18.2944  0.000000E+00       4.86893  0.000000E+00       4.86893  0.000000E+00  0.000000E+00      -9.73786       57.8987       1.83292        1       Scatag_D
       5       4431.17       9400.31  0.000000E+00  0.000000E+00       2540.98  0.000000E+00       7216.04       31029.3  0.000000E+00       19182.3       115.628       63497.9 -1.000000E+10 -1.000000E+10 -1.000000E+10 -1.000000E+10 -1.000000E+10  0.000000E+00  0.000000E+00  0.000000E+00  0.000000E+00  0.000000E+00  0.000000E+00  0.000000E+00  0.000000E+00  0.000000E+00  0.000000E+00  0.000000E+00  0.000000E+00       Funtag_D
       6  2.941562E+05  1.748483E+05  1.193079E+05       287.225     0.0551237      0.242978      0.207686      0.214319    -0.0184161    -0.0122028     0.0117640       1.43234       Opttag_D
       6       58.2947       2.78093       1.00000  0.000000E+00       1.00000  0.000000E+00      0.880576       64.3220       1.00000       18.4898  0.000000E+00       5.02394  0.000000E+00       5.02394  0.000000E+00  0.000000E+00      -10.0479       57.8987       1.83292        1       Scatag_D
       6       3890.71       11185.3  0.000000E+00  0.000000E+00       2533.02  0.000000E+00       5982.79       26134.4  0.000000E+00       17929.7       137.216       51514.8 -1.000000E+10 -1.000000E+10 -1.000000E+10 -1.000000E+10 -1.000000E+10  0.000000E+00  0.000000E+00  0.000000E+00  0.000000E+00  0.000000E+00  0.000000E+00  0.000000E+00  0.000000E+00  0.000000E+00  0.000000E+00  0.000000E+00  0.000000E+00       Funtag_D
       7  2.841198E+05  1.750247E+05  1.090951E+05       233.128     0.0615664      0.296303      0.208188      0.214844    -0.0197318    -0.0133185     0.0108660       1.38074       Opttag_D
       7       58.2485       2.79377       1.00000  0.000000E+00       1.00000  0.000000E+00      0.880439       64.0875       1.00000       18.5686  0.000000E+00       5.07611  0.000000E+00       5.07611  0.000000E+00  0.000000E+00      -10.1522       57.8987       1.83292        1       Scatag_D
       7       3319.36       10393.9  0.000000E+00  0.000000E+00       2530.54  0.000000E+00       5632.89       22226.3  0.000000E+00       17477.1       114.593       47400.4 -1.000000E+10 -1.000000E+10 -1.000000E+10 -1.000000E+10 -1.000000E+10  0.000000E+00  0.000000E+00  0.000000E+00  0.000000E+00  0.000000E+00  0.000000E+00  0.000000E+00  0.000000E+00  0.000000E+00  0.000000E+00  0.000000E+00  0.000000E+00       Funtag_D
       8  2.777654E+05  1.749773E+05  1.027881E+05       161.324     0.0627860      0.318489      0.208127      0.214915    -0.0193782    -0.0132289     0.0100078       1.30756       Opttag_D
       8       58.2220       2.78383       1.00000  0.000000E+00       1.00000  0.000000E+00      0.880329       64.0140       1.00000       18.5688  0.000000E+00       5.07757  0.000000E+00       5.07757  0.000000E+00  0.000000E+00      -10.1551       57.8987       1.83292        1       Scatag_D
       8       2815.78       9321.42  0.000000E+00  0.000000E+00       2527.51  0.000000E+00       5590.65       19387.4  0.000000E+00       17357.8       83.6924       45703.9 -1.000000E+10 -1.000000E+10 -1.000000E+10 -1.000000E+10 -1.000000E+10  0.000000E+00  0.000000E+00  0.000000E+00  0.000000E+00  0.000000E+00  0.000000E+00  0.000000E+00  0.000000E+00  0.000000E+00  0.000000E+00  0.000000E+00  0.000000E+00       Funtag_D
       9  2.696436E+05  1.750094E+05       94634.2       145.793     0.0680551      0.388442      0.208310      0.215426    -0.0196182    -0.0140176    0.00915532       1.25571       Opttag_D
       9       58.1598       2.77840       1.00000  0.000000E+00       1.00000  0.000000E+00      0.880157       63.7679       1.00000       18.5840  0.000000E+00       5.10998  0.000000E+00       5.10998  0.000000E+00  0.000000E+00      -10.2200       57.8987       1.83292        1       Scatag_D
       9       2356.49       8596.72  0.000000E+00  0.000000E+00       2521.14  0.000000E+00       5351.78       17371.4  0.000000E+00       16945.2       52.2240       41439.2 -1.000000E+10 -1.000000E+10 -1.000000E+10 -1.000000E+10 -1.000000E+10  0.000000E+00  0.000000E+00  0.000000E+00  0.000000E+00  0.000000E+00  0.000000E+00  0.000000E+00  0.000000E+00  0.000000E+00  0.000000E+00  0.000000E+00  0.000000E+00       Funtag_D
      10  2.646526E+05  1.751873E+05       89465.3       208.991     0.0755522      0.472527      0.208836      0.216208    -0.0209448    -0.0155508    0.00926885       1.31408       Opttag_D
      10       58.0969       2.78967       1.00000  0.000000E+00       1.00000  0.000000E+00      0.880044       63.4279       1.00000       18.6376  0.000000E+00       5.16810  0.000000E+00       5.16810  0.000000E+00  0.000000E+00      -10.3362       57.8987       1.83292        1       Scatag_D
      10       2415.30       9414.50  0.000000E+00  0.000000E+00       2516.68  0.000000E+00       4990.16       15697.2  0.000000E+00       16444.5       49.7634       37937.2 -1.000000E+10 -1.000000E+10 -1.000000E+10 -1.000000E+10 -1.000000E+10  0.000000E+00  0.000000E+00  0.000000E+00  0.000000E+00  0.000000E+00  0.000000E+00  0.000000E+00  0.000000E+00  0.000000E+00  0.000000E+00  0.000000E+00  0.000000E+00       Funtag_D
      11  2.597194E+05  1.752763E+05       84443.1       135.134     0.0795839      0.522003      0.209114      0.216633    -0.0216093    -0.0163164    0.00881138       1.23495       Opttag_D
      11       58.0567       2.79346       1.00000  0.000000E+00       1.00000  0.000000E+00      0.879977       63.2009       1.00000       18.6625  0.000000E+00       5.20427  0.000000E+00       5.20427  0.000000E+00  0.000000E+00      -10.4085       57.8987       1.83292        1       Scatag_D
      11       2182.76       8314.84  0.000000E+00  0.000000E+00       2512.75  0.000000E+00       4772.33       14293.0  0.000000E+00       16159.9       53.0371       36154.5 -1.000000E+10 -1.000000E+10 -1.000000E+10 -1.000000E+10 -1.000000E+10  0.000000E+00  0.000000E+00  0.000000E+00  0.000000E+00  0.000000E+00  0.000000E+00  0.000000E+00  0.000000E+00  0.000000E+00  0.000000E+00  0.000000E+00  0.000000E+00       Funtag_D
      12  2.543809E+05  1.754405E+05       78940.3       117.416     0.0856696      0.591330      0.209590      0.217229    -0.0228342    -0.0173993    0.00856283       1.20685       Opttag_D
      12       57.9910       2.80116       1.00000  0.000000E+00       1.00000  0.000000E+00      0.879878       62.8162       1.00000       18.6960  0.000000E+00       5.27107  0.000000E+00       5.27107  0.000000E+00  0.000000E+00      -10.5421       57.8987       1.83292        1       Scatag_D
      12       2061.36       7940.75  0.000000E+00  0.000000E+00       2507.82  0.000000E+00       4401.34       12429.6  0.000000E+00       15720.0       63.1915       33816.3 -1.000000E+10 -1.000000E+10 -1.000000E+10 -1.000000E+10 -1.000000E+10  0.000000E+00  0.000000E+00  0.000000E+00  0.000000E+00  0.000000E+00  0.000000E+00  0.000000E+00  0.000000E+00  0.000000E+00  0.000000E+00  0.000000E+00  0.000000E+00       Funtag_D
      13  2.512376E+05  1.755563E+05       75681.3       136.658     0.0896517      0.628593      0.209923      0.217546    -0.0236977    -0.0179982    0.00857210       1.21240       Opttag_D
      13       57.9392       2.80513       1.00000  0.000000E+00       1.00000  0.000000E+00      0.879772       62.5369       1.00000       18.7141  0.000000E+00       5.32230  0.000000E+00       5.32230  0.000000E+00  0.000000E+00      -10.6446       57.8987       1.83292        1       Scatag_D
      13       2065.82       8013.96  0.000000E+00  0.000000E+00       2503.67  0.000000E+00       4143.40       11160.7  0.000000E+00       15421.6       65.6879       32306.5 -1.000000E+10 -1.000000E+10 -1.000000E+10 -1.000000E+10 -1.000000E+10  0.000000E+00  0.000000E+00  0.000000E+00  0.000000E+00  0.000000E+00  0.000000E+00  0.000000E+00  0.000000E+00  0.000000E+00  0.000000E+00  0.000000E+00  0.000000E+00       Funtag_D
      14  2.479670E+05  1.756450E+05       72322.0       105.605     0.0927661      0.649443      0.210189      0.217767    -0.0243597    -0.0183058    0.00844309       1.17691       Opttag_D
      14       57.8882       2.80571       1.00000  0.000000E+00       1.00000  0.000000E+00      0.879644       62.2772       1.00000       18.7241  0.000000E+00       5.36870  0.000000E+00       5.36870  0.000000E+00  0.000000E+00      -10.7374       57.8987       1.83292        1       Scatag_D
      14       2004.11       7551.70  0.000000E+00  0.000000E+00       2499.62  0.000000E+00       3913.10       10252.3  0.000000E+00       15153.9       65.1485       30882.0 -1.000000E+10 -1.000000E+10 -1.000000E+10 -1.000000E+10 -1.000000E+10  0.000000E+00  0.000000E+00  0.000000E+00  0.000000E+00  0.000000E+00  0.000000E+00  0.000000E+00  0.000000E+00  0.000000E+00  0.000000E+00  0.000000E+00  0.000000E+00       Funtag_D
      15  2.450858E+05  1.757593E+05       69326.5       144.051     0.0968099      0.660529      0.210545      0.218149    -0.0252121    -0.0187924    0.00837310       1.18430       Opttag_D
      15       57.8131       2.80484       1.00000  0.000000E+00       1.00000  0.000000E+00      0.879420       61.9158       1.00000       18.7365  0.000000E+00       5.43316  0.000000E+00       5.43316  0.000000E+00  0.000000E+00      -10.8663       57.8987       1.83292        1       Scatag_D
      15       1971.02       7646.84  0.000000E+00  0.000000E+00       2495.64  0.000000E+00       3624.37       9735.30  0.000000E+00       14775.1       61.2642       29017.0 -1.000000E+10 -1.000000E+10 -1.000000E+10 -1.000000E+10 -1.000000E+10  0.000000E+00  0.000000E+00  0.000000E+00  0.000000E+00  0.000000E+00  0.000000E+00  0.000000E+00  0.000000E+00  0.000000E+00  0.000000E+00  0.000000E+00  0.000000E+00       Funtag_D
      16  2.425540E+05  1.757783E+05       66775.7       93.1310     0.0979347      0.651812      0.210632      0.218315    -0.0253543    -0.0189940    0.00802689       1.13826       Opttag_D
      16       57.7816       2.80091       1.00000  0.000000E+00       1.00000  0.000000E+00      0.879296       61.7762       1.00000       18.7424  0.000000E+00       5.45450  0.000000E+00       5.45450  0.000000E+00  0.000000E+00      -10.9090       57.8987       1.83292        1       Scatag_D
      16       1811.40       7063.84  0.000000E+00  0.000000E+00       2492.94  0.000000E+00       3531.67       9093.49  0.000000E+00       14610.6       55.4095       28116.3 -1.000000E+10 -1.000000E+10 -1.000000E+10 -1.000000E+10 -1.000000E+10  0.000000E+00  0.000000E+00  0.000000E+00  0.000000E+00  0.000000E+00  0.000000E+00  0.000000E+00  0.000000E+00  0.000000E+00  0.000000E+00  0.000000E+00  0.000000E+00       Funtag_D
      17  2.404244E+05  1.757880E+05       64636.5       85.5872     0.0989043      0.639798      0.210704      0.218484    -0.0254262    -0.0192937    0.00778821       1.12964       Opttag_D
      17       57.7538       2.79674       1.00000  0.000000E+00       1.00000  0.000000E+00      0.879180       61.6485       1.00000       18.7502  0.000000E+00       5.47241  0.000000E+00       5.47241  0.000000E+00  0.000000E+00      -10.9448       57.8987       1.83292        1       Scatag_D
      17       1705.27       6957.20  0.000000E+00  0.000000E+00       2490.46  0.000000E+00       3460.91       8267.73  0.000000E+00       14445.1       49.5898       27260.2 -1.000000E+10 -1.000000E+10 -1.000000E+10 -1.000000E+10 -1.000000E+10  0.000000E+00  0.000000E+00  0.000000E+00  0.000000E+00  0.000000E+00  0.000000E+00  0.000000E+00  0.000000E+00  0.000000E+00  0.000000E+00  0.000000E+00  0.000000E+00       Funtag_D
      18  2.378053E+05  1.758744E+05       61930.8       116.748      0.102322      0.625121      0.211027      0.219027    -0.0260714    -0.0204652    0.00766106       1.13497       Opttag_D
      18       57.6960       2.79622       1.00000  0.000000E+00       1.00000  0.000000E+00      0.878972       61.3265       1.00000       18.7813  0.000000E+00       5.51973  0.000000E+00       5.51973  0.000000E+00  0.000000E+00      -11.0395       57.8987       1.83292        1       Scatag_D
      18       1650.05       7022.98  0.000000E+00  0.000000E+00       2486.63  0.000000E+00       3319.23       7424.97  0.000000E+00       14059.9       47.9545       25919.1 -1.000000E+10 -1.000000E+10 -1.000000E+10 -1.000000E+10 -1.000000E+10  0.000000E+00  0.000000E+00  0.000000E+00  0.000000E+00  0.000000E+00  0.000000E+00  0.000000E+00  0.000000E+00  0.000000E+00  0.000000E+00  0.000000E+00  0.000000E+00       Funtag_D
      19  2.364057E+05  1.760464E+05       60359.3       143.696      0.107270      0.656560      0.211547      0.219704    -0.0273544    -0.0220919    0.00771320       1.14614       Opttag_D
      19       57.6391       2.80602       1.00000  0.000000E+00       1.00000  0.000000E+00      0.878801       60.9540       1.00000       18.8311  0.000000E+00       5.57993  0.000000E+00       5.57993  0.000000E+00  0.000000E+00      -11.1599       57.8987       1.83292        1       Scatag_D
      19       1672.58       7161.89  0.000000E+00  0.000000E+00       2481.52  0.000000E+00       3187.31       6851.10  0.000000E+00       13652.1       52.6298       25300.2 -1.000000E+10 -1.000000E+10 -1.000000E+10 -1.000000E+10 -1.000000E+10  0.000000E+00  0.000000E+00  0.000000E+00  0.000000E+00  0.000000E+00  0.000000E+00  0.000000E+00  0.000000E+00  0.000000E+00  0.000000E+00  0.000000E+00  0.000000E+00       Funtag_D
      20  2.348116E+05  1.760377E+05       58773.9       66.0651      0.107107      0.650311      0.211531      0.219671    -0.0272891    -0.0220123    0.00734387       1.08942       Opttag_D
      20       57.6417       2.80502       1.00000  0.000000E+00       1.00000  0.000000E+00      0.878816       60.9515       1.00000       18.8336  0.000000E+00       5.58054  0.000000E+00       5.58054  0.000000E+00  0.000000E+00      -11.1611       57.8987       1.83292        1       Scatag_D
      20       1516.24       6470.61  0.000000E+00  0.000000E+00       2481.08  0.000000E+00       3171.87       6468.99  0.000000E+00       13628.6       54.1209       24982.4 -1.000000E+10 -1.000000E+10 -1.000000E+10 -1.000000E+10 -1.000000E+10  0.000000E+00  0.000000E+00  0.000000E+00  0.000000E+00  0.000000E+00  0.000000E+00  0.000000E+00  0.000000E+00  0.000000E+00  0.000000E+00  0.000000E+00  0.000000E+00       Funtag_D
      21  2.335775E+05  1.760870E+05       57490.6       55.4158      0.108409      0.664976      0.211680      0.219807    -0.0276568    -0.0223817    0.00721254       1.07886       Opttag_D
      21       57.6294       2.80793       1.00000  0.000000E+00       1.00000  0.000000E+00      0.878789       60.8431       1.00000       18.8527  0.000000E+00       5.59992  0.000000E+00       5.59992  0.000000E+00  0.000000E+00      -11.1998       57.8987       1.83292        1       Scatag_D
      21       1462.50       6345.80  0.000000E+00  0.000000E+00       2479.08  0.000000E+00       3114.39       6063.94  0.000000E+00       13489.3       56.4004       24479.2 -1.000000E+10 -1.000000E+10 -1.000000E+10 -1.000000E+10 -1.000000E+10  0.000000E+00  0.000000E+00  0.000000E+00  0.000000E+00  0.000000E+00  0.000000E+00  0.000000E+00  0.000000E+00  0.000000E+00  0.000000E+00  0.000000E+00  0.000000E+00       Funtag_D
      22  2.324027E+05  1.761977E+05       56205.0       67.7417      0.111180      0.699322      0.212010      0.220115    -0.0284826    -0.0232317    0.00720856       1.07308       Opttag_D
      22       57.6012       2.81512       1.00000  0.000000E+00       1.00000  0.000000E+00      0.878705       60.6346       1.00000       18.8901  0.000000E+00       5.63658  0.000000E+00       5.63658  0.000000E+00  0.000000E+00      -11.2732       57.8987       1.83292        1       Scatag_D
      22       1460.88       6278.04  0.000000E+00  0.000000E+00       2475.64  0.000000E+00       3025.59       5755.09  0.000000E+00       13247.0       55.5586       23907.2 -1.000000E+10 -1.000000E+10 -1.000000E+10 -1.000000E+10 -1.000000E+10  0.000000E+00  0.000000E+00  0.000000E+00  0.000000E+00  0.000000E+00  0.000000E+00  0.000000E+00  0.000000E+00  0.000000E+00  0.000000E+00  0.000000E+00  0.000000E+00       Funtag_D
      23  2.312754E+05  1.764564E+05       54819.0       146.637      0.117261      0.755117      0.212749      0.220791    -0.0304126    -0.0251143    0.00745920       1.11471       Opttag_D
      23       57.5407       2.83345       1.00000  0.000000E+00       1.00000  0.000000E+00      0.878507       60.2021       1.00000       18.9790  0.000000E+00       5.71276  0.000000E+00       5.71276  0.000000E+00  0.000000E+00      -11.4255       57.8987       1.83292        1       Scatag_D
      23       1564.24       6774.51  0.000000E+00  0.000000E+00       2470.71  0.000000E+00       2860.16       5274.46  0.000000E+00       12759.3       47.0760       23068.5 -1.000000E+10 -1.000000E+10 -1.000000E+10 -1.000000E+10 -1.000000E+10  0.000000E+00  0.000000E+00  0.000000E+00  0.000000E+00  0.000000E+00  0.000000E+00  0.000000E+00  0.000000E+00  0.000000E+00  0.000000E+00  0.000000E+00  0.000000E+00       Funtag_D
      24  2.296833E+05  1.765337E+05       53149.6       56.0734      0.119094      0.767249      0.212975      0.221002    -0.0309895    -0.0256943    0.00713680       1.04647       Opttag_D
      24       57.5202       2.83849       1.00000  0.000000E+00       1.00000  0.000000E+00      0.878434       60.0678       1.00000       19.0097  0.000000E+00       5.73447  0.000000E+00       5.73447  0.000000E+00  0.000000E+00      -11.4689       57.8987       1.83292        1       Scatag_D
      24       1431.94       5970.48  0.000000E+00  0.000000E+00       2468.77  0.000000E+00       2813.65       4936.62  0.000000E+00       12611.5       43.7246       22872.9 -1.000000E+10 -1.000000E+10 -1.000000E+10 -1.000000E+10 -1.000000E+10  0.000000E+00  0.000000E+00  0.000000E+00  0.000000E+00  0.000000E+00  0.000000E+00  0.000000E+00  0.000000E+00  0.000000E+00  0.000000E+00  0.000000E+00  0.000000E+00       Funtag_D
      25  2.288408E+05  1.765599E+05       52280.9       49.2993      0.119939      0.764278      0.213067      0.221080    -0.0311847    -0.0259004    0.00694915       1.04191       Opttag_D
      25       57.5041       2.83835       1.00000  0.000000E+00       1.00000  0.000000E+00      0.878373       59.9875       1.00000       19.0258  0.000000E+00       5.74570  0.000000E+00       5.74570  0.000000E+00  0.000000E+00      -11.4914       57.8987       1.83292        1       Scatag_D
      25       1357.63       5918.59  0.000000E+00  0.000000E+00       2467.74  0.000000E+00       2778.40       4620.78  0.000000E+00       12505.3       40.3648       22592.1 -1.000000E+10 -1.000000E+10 -1.000000E+10 -1.000000E+10 -1.000000E+10  0.000000E+00  0.000000E+00  0.000000E+00  0.000000E+00  0.000000E+00  0.000000E+00  0.000000E+00  0.000000E+00  0.000000E+00  0.000000E+00  0.000000E+00  0.000000E+00       Funtag_D
      26  2.278795E+05  1.766340E+05       51245.5       56.3337      0.122082      0.763862      0.213303      0.221296    -0.0317378    -0.0265309    0.00681326       1.03889       Opttag_D
      26       57.4734       2.84081       1.00000  0.000000E+00       1.00000  0.000000E+00      0.878271       59.8033       1.00000       19.0641  0.000000E+00       5.77142  0.000000E+00       5.77142  0.000000E+00  0.000000E+00      -11.5428       57.8987       1.83292        1       Scatag_D
      26       1305.06       5884.35  0.000000E+00  0.000000E+00       2466.35  0.000000E+00       2712.39       4365.85  0.000000E+00       12289.6       39.8425       22182.0 -1.000000E+10 -1.000000E+10 -1.000000E+10 -1.000000E+10 -1.000000E+10  0.000000E+00  0.000000E+00  0.000000E+00  0.000000E+00  0.000000E+00  0.000000E+00  0.000000E+00  0.000000E+00  0.000000E+00  0.000000E+00  0.000000E+00  0.000000E+00       Funtag_D
      27  2.272379E+05  1.767884E+05       50449.5       147.430      0.126654      0.765402      0.213812      0.221791    -0.0328894    -0.0280455    0.00692350       1.09495       Opttag_D
      27       57.4188       2.84716       1.00000  0.000000E+00       1.00000  0.000000E+00      0.878112       59.4196       1.00000       19.1485  0.000000E+00       5.82210  0.000000E+00       5.82210  0.000000E+00  0.000000E+00      -11.6442       57.8987       1.83292        1       Scatag_D
      27       1347.63       6536.45  0.000000E+00  0.000000E+00       2463.94  0.000000E+00       2599.46       3977.61  0.000000E+00       11876.5       48.2404       21599.7 -1.000000E+10 -1.000000E+10 -1.000000E+10 -1.000000E+10 -1.000000E+10  0.000000E+00  0.000000E+00  0.000000E+00  0.000000E+00  0.000000E+00  0.000000E+00  0.000000E+00  0.000000E+00  0.000000E+00  0.000000E+00  0.000000E+00  0.000000E+00       Funtag_D
      28  2.259604E+05  1.768442E+05       49116.2       53.8979      0.128252      0.767139      0.213998      0.221971    -0.0333055    -0.0286086    0.00666335       1.02363       Opttag_D
      28       57.4026       2.85026       1.00000  0.000000E+00       1.00000  0.000000E+00      0.878069       59.2943       1.00000       19.1778  0.000000E+00       5.83832  0.000000E+00       5.83832  0.000000E+00  0.000000E+00      -11.6766       57.8987       1.83292        1       Scatag_D
      28       1248.26       5712.69  0.000000E+00  0.000000E+00       2463.88  0.000000E+00       2567.80       3866.31  0.000000E+00       11743.3       51.2242       21462.7 -1.000000E+10 -1.000000E+10 -1.000000E+10 -1.000000E+10 -1.000000E+10  0.000000E+00  0.000000E+00  0.000000E+00  0.000000E+00  0.000000E+00  0.000000E+00  0.000000E+00  0.000000E+00  0.000000E+00  0.000000E+00  0.000000E+00  0.000000E+00       Funtag_D
      29  2.253117E+05  1.768795E+05       48432.1       38.2257      0.129514      0.770999      0.214131      0.222098    -0.0335693    -0.0290888    0.00656086       1.01388       Opttag_D
      29       57.3912       2.85164       1.00000  0.000000E+00       1.00000  0.000000E+00      0.878046       59.1893       1.00000       19.2012  0.000000E+00       5.85125  0.000000E+00       5.85125  0.000000E+00  0.000000E+00      -11.7025       57.8987       1.83292        1       Scatag_D
      29       1210.15       5604.45  0.000000E+00  0.000000E+00       2463.36  0.000000E+00       2540.29       3677.10  0.000000E+00       11627.9       54.4966       21254.4 -1.000000E+10 -1.000000E+10 -1.000000E+10 -1.000000E+10 -1.000000E+10  0.000000E+00  0.000000E+00  0.000000E+00  0.000000E+00  0.000000E+00  0.000000E+00  0.000000E+00  0.000000E+00  0.000000E+00  0.000000E+00  0.000000E+00  0.000000E+00       Funtag_D
      30  2.245839E+05  1.769573E+05       47626.5       51.8938      0.132021      0.779651      0.214401      0.222360    -0.0341499    -0.0300774    0.00653253       1.00977       Opttag_D
      30       57.3711       2.85605       1.00000  0.000000E+00       1.00000  0.000000E+00      0.877999       58.9996       1.00000       19.2469  0.000000E+00       5.87544  0.000000E+00       5.87544  0.000000E+00  0.000000E+00      -11.7509       57.8987       1.83292        1       Scatag_D
      30       1199.72       5559.07  0.000000E+00  0.000000E+00       2462.47  0.000000E+00       2495.17       3500.23  0.000000E+00       11420.2       57.2456       20932.4 -1.000000E+10 -1.000000E+10 -1.000000E+10 -1.000000E+10 -1.000000E+10  0.000000E+00  0.000000E+00  0.000000E+00  0.000000E+00  0.000000E+00  0.000000E+00  0.000000E+00  0.000000E+00  0.000000E+00  0.000000E+00  0.000000E+00  0.000000E+00       Funtag_D
      31  2.237345E+05  1.771092E+05       46625.3       95.3163      0.136526      0.802682      0.214890      0.222893    -0.0352825    -0.0319030    0.00662651       1.02173       Opttag_D
      31       57.3409       2.86732       1.00000  0.000000E+00       1.00000  0.000000E+00      0.877922       58.7022       1.00000       19.3275  0.000000E+00       5.91433  0.000000E+00       5.91433  0.000000E+00  0.000000E+00      -11.8287       57.8987       1.83292        1       Scatag_D
      31       1234.49       5691.52  0.000000E+00  0.000000E+00       2461.67  0.000000E+00       2430.42       3215.11  0.000000E+00       11094.7       51.5360       20445.9 -1.000000E+10 -1.000000E+10 -1.000000E+10 -1.000000E+10 -1.000000E+10  0.000000E+00  0.000000E+00  0.000000E+00  0.000000E+00  0.000000E+00  0.000000E+00  0.000000E+00  0.000000E+00  0.000000E+00  0.000000E+00  0.000000E+00  0.000000E+00       Funtag_D
      32  2.227983E+05  1.772814E+05       45516.8       51.5556      0.141040      0.827353      0.215408      0.223490    -0.0365676    -0.0338161    0.00661828      0.988079       Opttag_D
      32       57.3173       2.88280       1.00000  0.000000E+00       1.00000  0.000000E+00      0.877869       58.4367       1.00000       19.4086  0.000000E+00       5.94915  0.000000E+00       5.94915  0.000000E+00  0.000000E+00      -11.8983       57.8987       1.83292        1       Scatag_D
      32       1231.43       5322.79  0.000000E+00  0.000000E+00       2462.14  0.000000E+00       2383.11       3082.86  0.000000E+00       10831.0       46.4698       20157.1 -1.000000E+10 -1.000000E+10 -1.000000E+10 -1.000000E+10 -1.000000E+10  0.000000E+00  0.000000E+00  0.000000E+00  0.000000E+00  0.000000E+00  0.000000E+00  0.000000E+00  0.000000E+00  0.000000E+00  0.000000E+00  0.000000E+00  0.000000E+00       Funtag_D
      33  2.221239E+05  1.773477E+05       44776.2       39.1058      0.142952      0.846368      0.215607      0.223753    -0.0370623    -0.0345319    0.00648532      0.980390       Opttag_D
      33       57.3078       2.88880       1.00000  0.000000E+00       1.00000  0.000000E+00      0.877849       58.3335       1.00000       19.4411  0.000000E+00       5.96224  0.000000E+00       5.96224  0.000000E+00  0.000000E+00      -11.9245       57.8987       1.83292        1       Scatag_D
      33       1182.45       5240.27  0.000000E+00  0.000000E+00       2461.97  0.000000E+00       2353.64       2919.39  0.000000E+00       10719.1       41.8657       19857.5 -1.000000E+10 -1.000000E+10 -1.000000E+10 -1.000000E+10 -1.000000E+10  0.000000E+00  0.000000E+00  0.000000E+00  0.000000E+00  0.000000E+00  0.000000E+00  0.000000E+00  0.000000E+00  0.000000E+00  0.000000E+00  0.000000E+00  0.000000E+00       Funtag_D
      34  2.213703E+05  1.774432E+05       43927.2       62.4424      0.145953      0.884549      0.215894      0.224193    -0.0377743    -0.0355647    0.00633415      0.985167       Opttag_D
      34       57.2940       2.89726       1.00000  0.000000E+00       1.00000  0.000000E+00      0.877836       58.1764       1.00000       19.4911  0.000000E+00       5.98085  0.000000E+00       5.98085  0.000000E+00  0.000000E+00      -11.9617       57.8987       1.83292        1       Scatag_D
      34       1127.96       5291.46  0.000000E+00  0.000000E+00       2461.17  0.000000E+00       2298.22       2813.90  0.000000E+00       10550.1       37.9094       19346.4 -1.000000E+10 -1.000000E+10 -1.000000E+10 -1.000000E+10 -1.000000E+10  0.000000E+00  0.000000E+00  0.000000E+00  0.000000E+00  0.000000E+00  0.000000E+00  0.000000E+00  0.000000E+00  0.000000E+00  0.000000E+00  0.000000E+00  0.000000E+00       Funtag_D
      35  2.208540E+05  1.774804E+05       43373.6       53.8571      0.147505      0.910221      0.216011      0.224393    -0.0380522    -0.0359691    0.00624175      0.978358       Opttag_D
      35       57.2875       2.90001       1.00000  0.000000E+00       1.00000  0.000000E+00      0.877849       58.0922       1.00000       19.5146  0.000000E+00       5.98971  0.000000E+00       5.98971  0.000000E+00  0.000000E+00      -11.9794       57.8987       1.83292        1       Scatag_D
      35       1095.30       5218.57  0.000000E+00  0.000000E+00       2459.68  0.000000E+00       2261.13       2765.43  0.000000E+00       10456.6       39.5115       19077.4 -1.000000E+10 -1.000000E+10 -1.000000E+10 -1.000000E+10 -1.000000E+10  0.000000E+00  0.000000E+00  0.000000E+00  0.000000E+00  0.000000E+00  0.000000E+00  0.000000E+00  0.000000E+00  0.000000E+00  0.000000E+00  0.000000E+00  0.000000E+00       Funtag_D
      36  2.204214E+05  1.774425E+05       42978.9       35.9398      0.147075      0.915421      0.215911      0.224299    -0.0377693    -0.0356501    0.00613779      0.971780       Opttag_D
      36       57.2885       2.89514       1.00000  0.000000E+00       1.00000  0.000000E+00      0.877871       58.1047       1.00000       19.5040  0.000000E+00       5.98691  0.000000E+00       5.98691  0.000000E+00  0.000000E+00      -11.9738       57.8987       1.83292        1       Scatag_D
      36       1059.11       5148.63  0.000000E+00  0.000000E+00       2458.55  0.000000E+00       2248.49       2671.67  0.000000E+00       10450.9       43.0345       18898.5 -1.000000E+10 -1.000000E+10 -1.000000E+10 -1.000000E+10 -1.000000E+10  0.000000E+00  0.000000E+00  0.000000E+00  0.000000E+00  0.000000E+00  0.000000E+00  0.000000E+00  0.000000E+00  0.000000E+00  0.000000E+00  0.000000E+00  0.000000E+00       Funtag_D
      37  2.198099E+05  1.774696E+05       42340.3       43.1559      0.148852      0.942368      0.216005      0.224546    -0.0379716    -0.0362102    0.00606501      0.969746       Opttag_D
      37       57.2848       2.89623       1.00000  0.000000E+00       1.00000  0.000000E+00      0.877938       57.9952       1.00000       19.5286  0.000000E+00       5.99831  0.000000E+00       5.99831  0.000000E+00  0.000000E+00      -11.9966       57.8987       1.83292        1       Scatag_D
      37       1034.14       5127.10  0.000000E+00  0.000000E+00       2455.82  0.000000E+00       2204.32       2528.89  0.000000E+00       10322.0       49.1673       18618.8 -1.000000E+10 -1.000000E+10 -1.000000E+10 -1.000000E+10 -1.000000E+10  0.000000E+00  0.000000E+00  0.000000E+00  0.000000E+00  0.000000E+00  0.000000E+00  0.000000E+00  0.000000E+00  0.000000E+00  0.000000E+00  0.000000E+00  0.000000E+00       Funtag_D
      38  2.195122E+05  1.775223E+05       41989.9       53.3426      0.151099      0.968265      0.216171      0.224900    -0.0383645    -0.0370636    0.00609582      0.970330       Opttag_D
      38       57.2820       2.90093       1.00000  0.000000E+00       1.00000  0.000000E+00      0.878013       57.8750       1.00000       19.5593  0.000000E+00       6.01170  0.000000E+00       6.01170  0.000000E+00  0.000000E+00      -12.0234       57.8987       1.83292        1       Scatag_D
      38       1044.68       5133.28  0.000000E+00  0.000000E+00       2453.15  0.000000E+00       2168.21       2481.39  0.000000E+00       10184.2       50.3934       18474.6 -1.000000E+10 -1.000000E+10 -1.000000E+10 -1.000000E+10 -1.000000E+10  0.000000E+00  0.000000E+00  0.000000E+00  0.000000E+00  0.000000E+00  0.000000E+00  0.000000E+00  0.000000E+00  0.000000E+00  0.000000E+00  0.000000E+00  0.000000E+00       Funtag_D
      39  2.192008E+05  1.775550E+05       41645.9       32.2596      0.152132      0.974837      0.216269      0.225077    -0.0386083    -0.0375471    0.00603513      0.960655       Opttag_D
      39       57.2810       2.90435       1.00000  0.000000E+00       1.00000  0.000000E+00      0.878038       57.8214       1.00000       19.5746  0.000000E+00       6.01793  0.000000E+00       6.01793  0.000000E+00  0.000000E+00      -12.0359       57.8987       1.83292        1       Scatag_D
      39       1023.98       5031.42  0.000000E+00  0.000000E+00       2452.67  0.000000E+00       2155.10       2412.74  0.000000E+00       10126.4       48.1631       18395.4 -1.000000E+10 -1.000000E+10 -1.000000E+10 -1.000000E+10 -1.000000E+10  0.000000E+00  0.000000E+00  0.000000E+00  0.000000E+00  0.000000E+00  0.000000E+00  0.000000E+00  0.000000E+00  0.000000E+00  0.000000E+00  0.000000E+00  0.000000E+00       Funtag_D
      40  2.186967E+05  1.776550E+05       41041.8       31.0337      0.155287      0.994272      0.216566      0.225608    -0.0393542    -0.0390238    0.00603361      0.955733       Opttag_D
      40       57.2789       2.91526       1.00000  0.000000E+00       1.00000  0.000000E+00      0.878127       57.6649       1.00000       19.6192  0.000000E+00       6.03576  0.000000E+00       6.03576  0.000000E+00  0.000000E+00      -12.0715       57.8987       1.83292        1       Scatag_D
      40       1023.47       4980.00  0.000000E+00  0.000000E+00       2451.54  0.000000E+00       2117.20       2292.04  0.000000E+00       9955.38       41.7330       18180.4 -1.000000E+10 -1.000000E+10 -1.000000E+10 -1.000000E+10 -1.000000E+10  0.000000E+00  0.000000E+00  0.000000E+00  0.000000E+00  0.000000E+00  0.000000E+00  0.000000E+00  0.000000E+00  0.000000E+00  0.000000E+00  0.000000E+00  0.000000E+00       Funtag_D
      41  2.183265E+05  1.777251E+05       40601.4       35.3677      0.157793       1.01046      0.216780      0.226009    -0.0398772    -0.0401413    0.00601568      0.954635       Opttag_D
      41       57.2783       2.92295       1.00000  0.000000E+00       1.00000  0.000000E+00      0.878221       57.5428       1.00000       19.6512  0.000000E+00       6.04818  0.000000E+00       6.04818  0.000000E+00  0.000000E+00      -12.0964       57.8987       1.83292        1       Scatag_D
      41       1017.39       4968.56  0.000000E+00  0.000000E+00       2450.89  0.000000E+00       2084.98       2222.43  0.000000E+00       9813.88       39.0224       18004.2 -1.000000E+10 -1.000000E+10 -1.000000E+10 -1.000000E+10 -1.000000E+10  0.000000E+00  0.000000E+00  0.000000E+00  0.000000E+00  0.000000E+00  0.000000E+00  0.000000E+00  0.000000E+00  0.000000E+00  0.000000E+00  0.000000E+00  0.000000E+00       Funtag_D
      42  2.180214E+05  1.779178E+05       40103.5       85.9592      0.164495       1.06924      0.217363      0.227054    -0.0413153    -0.0431202    0.00613903      0.978093       Opttag_D
      42       57.2831       2.94534       1.00000  0.000000E+00       1.00000  0.000000E+00      0.878504       57.2340       1.00000       19.7383  0.000000E+00       6.07712  0.000000E+00       6.07712  0.000000E+00  0.000000E+00      -12.1542       57.8987       1.83292        1       Scatag_D
      42       1059.54       5215.74  0.000000E+00  0.000000E+00       2450.59  0.000000E+00       2011.65       2130.50  0.000000E+00       9470.62       36.7266       17728.1 -1.000000E+10 -1.000000E+10 -1.000000E+10 -1.000000E+10 -1.000000E+10  0.000000E+00  0.000000E+00  0.000000E+00  0.000000E+00  0.000000E+00  0.000000E+00  0.000000E+00  0.000000E+00  0.000000E+00  0.000000E+00  0.000000E+00  0.000000E+00       Funtag_D
      43  2.174625E+05  1.779098E+05       39552.7       27.0015      0.164692       1.07218      0.217353      0.227068    -0.0412555    -0.0431485    0.00592661      0.949784       Opttag_D
      43       57.2840       2.94411       1.00000  0.000000E+00       1.00000  0.000000E+00      0.878531       57.2192       1.00000       19.7386  0.000000E+00       6.07727  0.000000E+00       6.07727  0.000000E+00  0.000000E+00      -12.1545       57.8987       1.83292        1       Scatag_D
      43       987.486       4918.19  0.000000E+00  0.000000E+00       2450.19  0.000000E+00       2004.24       2064.80  0.000000E+00       9441.52       38.5669       17647.7 -1.000000E+10 -1.000000E+10 -1.000000E+10 -1.000000E+10 -1.000000E+10  0.000000E+00  0.000000E+00  0.000000E+00  0.000000E+00  0.000000E+00  0.000000E+00  0.000000E+00  0.000000E+00  0.000000E+00  0.000000E+00  0.000000E+00  0.000000E+00       Funtag_D
      44  2.172743E+05  1.778829E+05       39391.3       22.8717      0.164310       1.06901      0.217288      0.226993    -0.0410550    -0.0429049    0.00586519      0.948585       Opttag_D
      44       57.2847       2.94058       1.00000  0.000000E+00       1.00000  0.000000E+00      0.878532       57.2283       1.00000       19.7315  0.000000E+00       6.07507  0.000000E+00       6.07507  0.000000E+00  0.000000E+00      -12.1501       57.8987       1.83292        1       Scatag_D
      44       967.127       4905.78  0.000000E+00  0.000000E+00       2449.60  0.000000E+00       2001.96       2025.30  0.000000E+00       9437.93       40.8469       17562.8 -1.000000E+10 -1.000000E+10 -1.000000E+10 -1.000000E+10 -1.000000E+10  0.000000E+00  0.000000E+00  0.000000E+00  0.000000E+00  0.000000E+00  0.000000E+00  0.000000E+00  0.000000E+00  0.000000E+00  0.000000E+00  0.000000E+00  0.000000E+00       Funtag_D
      45  2.169445E+05  1.778860E+05       39058.5       28.3178      0.165399       1.08069      0.217326      0.227150    -0.0410776    -0.0432580    0.00581747      0.947741       Opttag_D
      45       57.2906       2.94043       1.00000  0.000000E+00       1.00000  0.000000E+00      0.878617       57.1645       1.00000       19.7456  0.000000E+00       6.07826  0.000000E+00       6.07826  0.000000E+00  0.000000E+00      -12.1565       57.8987       1.83292        1       Scatag_D
      45       951.452       4897.06  0.000000E+00  0.000000E+00       2448.18  0.000000E+00       1984.21       2004.64  0.000000E+00       9350.49       44.3627       17378.2 -1.000000E+10 -1.000000E+10 -1.000000E+10 -1.000000E+10 -1.000000E+10  0.000000E+00  0.000000E+00  0.000000E+00  0.000000E+00  0.000000E+00  0.000000E+00  0.000000E+00  0.000000E+00  0.000000E+00  0.000000E+00  0.000000E+00  0.000000E+00       Funtag_D
      46  2.167123E+05  1.779575E+05       38754.8       76.0976      0.169147       1.11613      0.217568      0.227751    -0.0416114    -0.0447371    0.00592340      0.966662       Opttag_D
      46       57.3083       2.94912       1.00000  0.000000E+00       1.00000  0.000000E+00      0.878837       56.9873       1.00000       19.8018  0.000000E+00       6.09028  0.000000E+00       6.09028  0.000000E+00  0.000000E+00      -12.1806       57.8987       1.83292        1       Scatag_D
      46       986.416       5094.54  0.000000E+00  0.000000E+00       2445.00  0.000000E+00       1951.41       1984.26  0.000000E+00       9147.54       43.9300       17101.7 -1.000000E+10 -1.000000E+10 -1.000000E+10 -1.000000E+10 -1.000000E+10  0.000000E+00  0.000000E+00  0.000000E+00  0.000000E+00  0.000000E+00  0.000000E+00  0.000000E+00  0.000000E+00  0.000000E+00  0.000000E+00  0.000000E+00  0.000000E+00       Funtag_D
      47  2.162721E+05  1.779956E+05       38276.5       34.3829      0.170689       1.12987      0.217689      0.228014    -0.0418951    -0.0453797    0.00578938      0.943689       Opttag_D
      47       57.3154       2.95388       1.00000  0.000000E+00       1.00000  0.000000E+00      0.878919       56.9186       1.00000       19.8271  0.000000E+00       6.09525  0.000000E+00       6.09525  0.000000E+00  0.000000E+00      -12.1905       57.8987       1.83292        1       Scatag_D
      47       942.285       4855.27  0.000000E+00  0.000000E+00       2444.68  0.000000E+00       1940.55       1959.56  0.000000E+00       9080.01       43.0697       17011.1 -1.000000E+10 -1.000000E+10 -1.000000E+10 -1.000000E+10 -1.000000E+10  0.000000E+00  0.000000E+00  0.000000E+00  0.000000E+00  0.000000E+00  0.000000E+00  0.000000E+00  0.000000E+00  0.000000E+00  0.000000E+00  0.000000E+00  0.000000E+00       Funtag_D
      48  2.159128E+05  1.780793E+05       37833.5       25.5407      0.173671       1.15543      0.217936      0.228515    -0.0425199    -0.0466529    0.00575812      0.938601       Opttag_D
      48       57.3290       2.96479       1.00000  0.000000E+00       1.00000  0.000000E+00      0.879064       56.7951       1.00000       19.8766  0.000000E+00       6.10465  0.000000E+00       6.10465  0.000000E+00  0.000000E+00      -12.2093       57.8987       1.83292        1       Scatag_D
      48       932.137       4803.06  0.000000E+00  0.000000E+00       2444.37  0.000000E+00       1921.06       1889.27  0.000000E+00       8964.42       39.4547       16839.7 -1.000000E+10 -1.000000E+10 -1.000000E+10 -1.000000E+10 -1.000000E+10  0.000000E+00  0.000000E+00  0.000000E+00  0.000000E+00  0.000000E+00  0.000000E+00  0.000000E+00  0.000000E+00  0.000000E+00  0.000000E+00  0.000000E+00  0.000000E+00       Funtag_D
      49  2.156019E+05  1.781760E+05       37425.9       31.6007      0.177050       1.18308      0.218219      0.229072    -0.0432415    -0.0480801    0.00574524      0.936035       Opttag_D
      49       57.3459       2.97785       1.00000  0.000000E+00       1.00000  0.000000E+00      0.879230       56.6625       1.00000       19.9321  0.000000E+00       6.11412  0.000000E+00       6.11412  0.000000E+00  0.000000E+00      -12.2282       57.8987       1.83292        1       Scatag_D
      49       927.972       4776.83  0.000000E+00  0.000000E+00       2444.24  0.000000E+00       1898.94       1855.19  0.000000E+00       8842.82       36.4333       16643.4 -1.000000E+10 -1.000000E+10 -1.000000E+10 -1.000000E+10 -1.000000E+10  0.000000E+00  0.000000E+00  0.000000E+00  0.000000E+00  0.000000E+00  0.000000E+00  0.000000E+00  0.000000E+00  0.000000E+00  0.000000E+00  0.000000E+00  0.000000E+00       Funtag_D
      50  2.152052E+05  1.783119E+05       36893.3       60.8232      0.181761       1.21906      0.218611      0.229818    -0.0442554    -0.0499885    0.00570517      0.937245       Opttag_D
      50       57.3725       2.99718       1.00000  0.000000E+00       1.00000  0.000000E+00      0.879457       56.4900       1.00000       20.0032  0.000000E+00       6.12558  0.000000E+00       6.12558  0.000000E+00  0.000000E+00      -12.2512       57.8987       1.83292        1       Scatag_D
      50       915.071       4789.19  0.000000E+00  0.000000E+00       2444.38  0.000000E+00       1860.67       1852.28  0.000000E+00       8679.53       36.5916       16315.6 -1.000000E+10 -1.000000E+10 -1.000000E+10 -1.000000E+10 -1.000000E+10  0.000000E+00  0.000000E+00  0.000000E+00  0.000000E+00  0.000000E+00  0.000000E+00  0.000000E+00  0.000000E+00  0.000000E+00  0.000000E+00  0.000000E+00  0.000000E+00       Funtag_D
      51  2.148429E+05  1.783989E+05       36443.9       32.9549      0.184767       1.24048      0.218853      0.230290    -0.0449042    -0.0512186    0.00565406      0.921573       Opttag_D
      51       57.3920       3.01006       1.00000  0.000000E+00       1.00000  0.000000E+00      0.879609       56.3848       1.00000       20.0463  0.000000E+00       6.13252  0.000000E+00       6.13252  0.000000E+00  0.000000E+00      -12.2650       57.8987       1.83292        1       Scatag_D
      51       898.751       4630.37  0.000000E+00  0.000000E+00       2443.57  0.000000E+00       1839.62       1824.83  0.000000E+00       8581.28       37.4108       16188.1 -1.000000E+10 -1.000000E+10 -1.000000E+10 -1.000000E+10 -1.000000E+10  0.000000E+00  0.000000E+00  0.000000E+00  0.000000E+00  0.000000E+00  0.000000E+00  0.000000E+00  0.000000E+00  0.000000E+00  0.000000E+00  0.000000E+00  0.000000E+00       Funtag_D
      52  2.145950E+05  1.783334E+05       36261.6       20.2158      0.183079       1.22671      0.218670      0.229973    -0.0444157    -0.0504169    0.00555675      0.917690       Opttag_D
      52       57.3849       3.00132       1.00000  0.000000E+00       1.00000  0.000000E+00      0.879527       56.4456       1.00000       20.0103  0.000000E+00       6.12817  0.000000E+00       6.12817  0.000000E+00  0.000000E+00      -12.2563       57.8987       1.83292        1       Scatag_D
      52       868.082       4591.43  0.000000E+00  0.000000E+00       2442.05  0.000000E+00       1843.66       1779.29  0.000000E+00       8615.59       39.1077       16082.4 -1.000000E+10 -1.000000E+10 -1.000000E+10 -1.000000E+10 -1.000000E+10  0.000000E+00  0.000000E+00  0.000000E+00  0.000000E+00  0.000000E+00  0.000000E+00  0.000000E+00  0.000000E+00  0.000000E+00  0.000000E+00  0.000000E+00  0.000000E+00       Funtag_D
      53  2.143824E+05  1.783028E+05       36079.5       23.4519      0.182606       1.22129      0.218582      0.229837    -0.0441873    -0.0500603    0.00551827      0.914703       Opttag_D
      53       57.3876       2.99797       1.00000  0.000000E+00       1.00000  0.000000E+00      0.879515       56.4627       1.00000       19.9916  0.000000E+00       6.12712  0.000000E+00       6.12712  0.000000E+00  0.000000E+00      -12.2542       57.8987       1.83292        1       Scatag_D
      53       856.100       4561.59  0.000000E+00  0.000000E+00       2439.74  0.000000E+00       1843.15       1755.68  0.000000E+00       8610.13       39.7031       15973.4 -1.000000E+10 -1.000000E+10 -1.000000E+10 -1.000000E+10 -1.000000E+10  0.000000E+00  0.000000E+00  0.000000E+00  0.000000E+00  0.000000E+00  0.000000E+00  0.000000E+00  0.000000E+00  0.000000E+00  0.000000E+00  0.000000E+00  0.000000E+00       Funtag_D
      54  2.142089E+05  1.783169E+05       35892.0       36.3745      0.183810       1.22707      0.218620      0.229973    -0.0442920    -0.0504133    0.00555117      0.910853       Opttag_D
      54       57.4042       3.00160       1.00000  0.000000E+00       1.00000  0.000000E+00      0.879601       56.4191       1.00000       19.9955  0.000000E+00       6.12975  0.000000E+00       6.12975  0.000000E+00  0.000000E+00      -12.2595       57.8987       1.83292        1       Scatag_D
      54       866.337       4523.27  0.000000E+00  0.000000E+00       2436.53  0.000000E+00       1838.15       1767.11  0.000000E+00       8549.21       38.8180       15872.6 -1.000000E+10 -1.000000E+10 -1.000000E+10 -1.000000E+10 -1.000000E+10  0.000000E+00  0.000000E+00  0.000000E+00  0.000000E+00  0.000000E+00  0.000000E+00  0.000000E+00  0.000000E+00  0.000000E+00  0.000000E+00  0.000000E+00  0.000000E+00       Funtag_D
      55  2.140147E+05  1.783349E+05       35679.8       18.1574      0.184591       1.23213      0.218671      0.230081    -0.0444265    -0.0506753    0.00552688      0.906195       Opttag_D
      55       57.4124       3.00466       1.00000  0.000000E+00       1.00000  0.000000E+00      0.879652       56.3888       1.00000       20.0056  0.000000E+00       6.13176  0.000000E+00       6.13176  0.000000E+00  0.000000E+00      -12.2635       57.8987       1.83292        1       Scatag_D
      55       858.775       4477.13  0.000000E+00  0.000000E+00       2435.86  0.000000E+00       1834.75       1709.06  0.000000E+00       8518.03       37.3154       15808.9 -1.000000E+10 -1.000000E+10 -1.000000E+10 -1.000000E+10 -1.000000E+10  0.000000E+00  0.000000E+00  0.000000E+00  0.000000E+00  0.000000E+00  0.000000E+00  0.000000E+00  0.000000E+00  0.000000E+00  0.000000E+00  0.000000E+00  0.000000E+00       Funtag_D
      56  2.138203E+05  1.783782E+05       35442.1       20.1383      0.186276       1.24339      0.218794      0.230331    -0.0447494    -0.0513013    0.00553147      0.902931       Opttag_D
      56       57.4287       3.01172       1.00000  0.000000E+00       1.00000  0.000000E+00      0.879759       56.3254       1.00000       20.0290  0.000000E+00       6.13563  0.000000E+00       6.13563  0.000000E+00  0.000000E+00      -12.2713       57.8987       1.83292        1       Scatag_D
      56       860.200       4444.93  0.000000E+00  0.000000E+00       2435.26  0.000000E+00       1826.75       1687.51  0.000000E+00       8456.34       35.1351       15696.0 -1.000000E+10 -1.000000E+10 -1.000000E+10 -1.000000E+10 -1.000000E+10  0.000000E+00  0.000000E+00  0.000000E+00  0.000000E+00  0.000000E+00  0.000000E+00  0.000000E+00  0.000000E+00  0.000000E+00  0.000000E+00  0.000000E+00  0.000000E+00       Funtag_D
      57  2.136363E+05  1.784165E+05       35219.8       24.5948      0.187933       1.25350      0.218896      0.230555    -0.0450354    -0.0519263    0.00551043      0.900157       Opttag_D
      57       57.4470       3.01868       1.00000  0.000000E+00       1.00000  0.000000E+00      0.879867       56.2676       1.00000       20.0466  0.000000E+00       6.13870  0.000000E+00       6.13870  0.000000E+00  0.000000E+00      -12.2774       57.8987       1.83292        1       Scatag_D
      57       853.669       4417.66  0.000000E+00  0.000000E+00       2434.92  0.000000E+00       1816.57       1676.34  0.000000E+00       8392.06       34.7715       15593.8 -1.000000E+10 -1.000000E+10 -1.000000E+10 -1.000000E+10 -1.000000E+10  0.000000E+00  0.000000E+00  0.000000E+00  0.000000E+00  0.000000E+00  0.000000E+00  0.000000E+00  0.000000E+00  0.000000E+00  0.000000E+00  0.000000E+00  0.000000E+00       Funtag_D
      58  2.135244E+05  1.785172E+05       35007.2       58.5244      0.192183       1.27683      0.219160      0.231143    -0.0457869    -0.0535563    0.00551284      0.901450       Opttag_D
      58       57.4988       3.03808       1.00000  0.000000E+00       1.00000  0.000000E+00      0.880150       56.1360       1.00000       20.0842  0.000000E+00       6.14516  0.000000E+00       6.14516  0.000000E+00  0.000000E+00      -12.2903       57.8987       1.83292        1       Scatag_D
      58       854.414       4430.37  0.000000E+00  0.000000E+00       2434.68  0.000000E+00       1790.50       1761.35  0.000000E+00       8234.51       35.4258       15466.0 -1.000000E+10 -1.000000E+10 -1.000000E+10 -1.000000E+10 -1.000000E+10  0.000000E+00  0.000000E+00  0.000000E+00  0.000000E+00  0.000000E+00  0.000000E+00  0.000000E+00  0.000000E+00  0.000000E+00  0.000000E+00  0.000000E+00  0.000000E+00       Funtag_D
      59  2.132181E+05  1.785085E+05       34709.6       18.4038      0.192053       1.27543      0.219138      0.231122    -0.0457220    -0.0535461    0.00540799      0.892715       Opttag_D
      59       57.4997       3.03729       1.00000  0.000000E+00       1.00000  0.000000E+00      0.880145       56.1426       1.00000       20.0775  0.000000E+00       6.14458  0.000000E+00       6.14458  0.000000E+00  0.000000E+00      -12.2892       57.8987       1.83292        1       Scatag_D
      59       822.223       4344.92  0.000000E+00  0.000000E+00       2435.00  0.000000E+00       1786.33       1624.00  0.000000E+00       8230.98       36.2251       15429.9 -1.000000E+10 -1.000000E+10 -1.000000E+10 -1.000000E+10 -1.000000E+10  0.000000E+00  0.000000E+00  0.000000E+00  0.000000E+00  0.000000E+00  0.000000E+00  0.000000E+00  0.000000E+00  0.000000E+00  0.000000E+00  0.000000E+00  0.000000E+00       Funtag_D
      60  2.131146E+05  1.784975E+05       34617.1       14.9787      0.191878       1.27345      0.219106      0.231095    -0.0456394    -0.0535161    0.00536331      0.891940       Opttag_D
      60       57.5011       3.03629       1.00000  0.000000E+00       1.00000  0.000000E+00      0.880140       56.1516       1.00000       20.0685  0.000000E+00       6.14383  0.000000E+00       6.14383  0.000000E+00  0.000000E+00      -12.2877       57.8987       1.83292        1       Scatag_D
      60       808.693       4337.38  0.000000E+00  0.000000E+00       2434.83  0.000000E+00       1781.46       1601.79  0.000000E+00       8227.28       36.9197       15388.8 -1.000000E+10 -1.000000E+10 -1.000000E+10 -1.000000E+10 -1.000000E+10  0.000000E+00  0.000000E+00  0.000000E+00  0.000000E+00  0.000000E+00  0.000000E+00  0.000000E+00  0.000000E+00  0.000000E+00  0.000000E+00  0.000000E+00  0.000000E+00       Funtag_D
      61  2.129613E+05  1.785072E+05       34454.1       18.0601      0.192757       1.27589      0.219127      0.231220    -0.0457118    -0.0539094    0.00534539      0.891020       Opttag_D
      61       57.5187       3.04023       1.00000  0.000000E+00       1.00000  0.000000E+00      0.880212       56.1336       1.00000       20.0642  0.000000E+00       6.14430  0.000000E+00       6.14430  0.000000E+00  0.000000E+00      -12.2886       57.8987       1.83292        1       Scatag_D
      61       803.299       4328.44  0.000000E+00  0.000000E+00       2434.16  0.000000E+00       1767.63       1590.64  0.000000E+00       8183.72       37.2828       15308.9 -1.000000E+10 -1.000000E+10 -1.000000E+10 -1.000000E+10 -1.000000E+10  0.000000E+00  0.000000E+00  0.000000E+00  0.000000E+00  0.000000E+00  0.000000E+00  0.000000E+00  0.000000E+00  0.000000E+00  0.000000E+00  0.000000E+00  0.000000E+00       Funtag_D
      62  2.129251E+05  1.785495E+05       34375.7       55.0029      0.195279       1.28424      0.219223      0.231612    -0.0460274    -0.0549852    0.00545409      0.895128       Opttag_D
      62       57.5617       3.05200       1.00000  0.000000E+00       1.00000  0.000000E+00      0.880401       56.0785       1.00000       20.0701  0.000000E+00       6.14605  0.000000E+00       6.14605  0.000000E+00  0.000000E+00      -12.2921       57.8987       1.83292        1       Scatag_D
      62       836.303       4368.43  0.000000E+00  0.000000E+00       2431.50  0.000000E+00       1745.15       1678.42  0.000000E+00       8091.58       36.0009       15188.3 -1.000000E+10 -1.000000E+10 -1.000000E+10 -1.000000E+10 -1.000000E+10  0.000000E+00  0.000000E+00  0.000000E+00  0.000000E+00  0.000000E+00  0.000000E+00  0.000000E+00  0.000000E+00  0.000000E+00  0.000000E+00  0.000000E+00  0.000000E+00       Funtag_D
      63  2.127068E+05  1.785560E+05       34150.8       19.0328      0.195598       1.28543      0.219240      0.231642    -0.0460765    -0.0550684    0.00539860      0.888186       Opttag_D
      63       57.5664       3.05352       1.00000  0.000000E+00       1.00000  0.000000E+00      0.880423       56.0708       1.00000       20.0722  0.000000E+00       6.14628  0.000000E+00       6.14628  0.000000E+00  0.000000E+00      -12.2926       57.8987       1.83292        1       Scatag_D
      63       819.371       4300.94  0.000000E+00  0.000000E+00       2431.00  0.000000E+00       1742.90       1570.00  0.000000E+00       8081.05       35.8959       15169.6 -1.000000E+10 -1.000000E+10 -1.000000E+10 -1.000000E+10 -1.000000E+10  0.000000E+00  0.000000E+00  0.000000E+00  0.000000E+00  0.000000E+00  0.000000E+00  0.000000E+00  0.000000E+00  0.000000E+00  0.000000E+00  0.000000E+00  0.000000E+00       Funtag_D
      64  2.126108E+05  1.785738E+05       34037.0       12.6435      0.196447       1.28867      0.219285      0.231754    -0.0462087    -0.0553581    0.00539101      0.886323       Opttag_D
      64       57.5786       3.05753       1.00000  0.000000E+00       1.00000  0.000000E+00      0.880481       56.0511       1.00000       20.0784  0.000000E+00       6.14698  0.000000E+00       6.14698  0.000000E+00  0.000000E+00      -12.2940       57.8987       1.83292        1       Scatag_D
      64       817.069       4282.92  0.000000E+00  0.000000E+00       2429.72  0.000000E+00       1738.20       1549.25  0.000000E+00       8055.54       35.3518       15128.9 -1.000000E+10 -1.000000E+10 -1.000000E+10 -1.000000E+10 -1.000000E+10  0.000000E+00  0.000000E+00  0.000000E+00  0.000000E+00  0.000000E+00  0.000000E+00  0.000000E+00  0.000000E+00  0.000000E+00  0.000000E+00  0.000000E+00  0.000000E+00       Funtag_D
      65  2.124821E+05  1.786141E+05       33868.0       18.7868      0.198405       1.29613      0.219385      0.232020    -0.0465092    -0.0560689    0.00540814      0.884367       Opttag_D
      65       57.6082       3.06711       1.00000  0.000000E+00       1.00000  0.000000E+00      0.880615       56.0112       1.00000       20.0899  0.000000E+00       6.14803  0.000000E+00       6.14803  0.000000E+00  0.000000E+00      -12.2961       57.8987       1.83292        1       Scatag_D
      65       822.269       4264.04  0.000000E+00  0.000000E+00       2426.52  0.000000E+00       1727.41       1546.36  0.000000E+00       7998.87       34.7613       15047.8 -1.000000E+10 -1.000000E+10 -1.000000E+10 -1.000000E+10 -1.000000E+10  0.000000E+00  0.000000E+00  0.000000E+00  0.000000E+00  0.000000E+00  0.000000E+00  0.000000E+00  0.000000E+00  0.000000E+00  0.000000E+00  0.000000E+00  0.000000E+00       Funtag_D
      66  2.123138E+05  1.786689E+05       33645.0       26.2631      0.201067       1.33050      0.219516      0.232369    -0.0469183    -0.0570449    0.00536869      0.883113       Opttag_D
      66       57.6495       3.08072       1.00000  0.000000E+00       1.00000  0.000000E+00      0.880789       55.9686       1.00000       20.1007  0.000000E+00       6.14861  0.000000E+00       6.14861  0.000000E+00  0.000000E+00      -12.2972       57.8987       1.83292        1       Scatag_D
      66       810.319       4251.95  0.000000E+00  0.000000E+00       2420.87  0.000000E+00       1712.65       1545.69  0.000000E+00       7924.92       35.3266       14943.2 -1.000000E+10 -1.000000E+10 -1.000000E+10 -1.000000E+10 -1.000000E+10  0.000000E+00  0.000000E+00  0.000000E+00  0.000000E+00  0.000000E+00  0.000000E+00  0.000000E+00  0.000000E+00  0.000000E+00  0.000000E+00  0.000000E+00  0.000000E+00       Funtag_D
      67  2.122101E+05  1.787796E+05       33430.4       33.7818      0.206053       1.39185      0.219784      0.233032    -0.0477444    -0.0589440    0.00535351      0.881945       Opttag_D
      67       57.7281       3.10797       1.00000  0.000000E+00       1.00000  0.000000E+00      0.881108       55.9023       1.00000       20.1246  0.000000E+00       6.14812  0.000000E+00       6.14812  0.000000E+00  0.000000E+00      -12.2962       57.8987       1.83292        1       Scatag_D
      67       805.740       4240.71  0.000000E+00  0.000000E+00       2410.97  0.000000E+00       1691.40       1584.37  0.000000E+00       7808.58       35.8410       14852.8 -1.000000E+10 -1.000000E+10 -1.000000E+10 -1.000000E+10 -1.000000E+10  0.000000E+00  0.000000E+00  0.000000E+00  0.000000E+00  0.000000E+00  0.000000E+00  0.000000E+00  0.000000E+00  0.000000E+00  0.000000E+00  0.000000E+00  0.000000E+00       Funtag_D
      68  2.120531E+05  1.787611E+05       33292.0       13.4517      0.205515       1.38491      0.219735      0.232962    -0.0476065    -0.0587505    0.00528262      0.879940       Opttag_D
      68       57.7226       3.10486       1.00000  0.000000E+00       1.00000  0.000000E+00      0.881069       55.9178       1.00000       20.1163  0.000000E+00       6.14720  0.000000E+00       6.14720  0.000000E+00  0.000000E+00      -12.2944       57.8987       1.83292        1       Scatag_D
      68       784.543       4221.45  0.000000E+00  0.000000E+00       2410.12  0.000000E+00       1690.93       1513.57  0.000000E+00       7814.74       35.6608       14821.0 -1.000000E+10 -1.000000E+10 -1.000000E+10 -1.000000E+10 -1.000000E+10  0.000000E+00  0.000000E+00  0.000000E+00  0.000000E+00  0.000000E+00  0.000000E+00  0.000000E+00  0.000000E+00  0.000000E+00  0.000000E+00  0.000000E+00  0.000000E+00       Funtag_D
      69  2.119748E+05  1.787471E+05       33227.7       14.1526      0.205261       1.38116      0.219696      0.232926    -0.0475018    -0.0586600    0.00524401      0.879070       Opttag_D
      69       57.7225       3.10324       1.00000  0.000000E+00       1.00000  0.000000E+00      0.881047       55.9306       1.00000       20.1094  0.000000E+00       6.14592  0.000000E+00       6.14592  0.000000E+00  0.000000E+00      -12.2918       57.8987       1.83292        1       Scatag_D
      69       773.118       4213.11  0.000000E+00  0.000000E+00       2408.50  0.000000E+00       1689.29       1508.55  0.000000E+00       7814.27       35.3575       14785.5 -1.000000E+10 -1.000000E+10 -1.000000E+10 -1.000000E+10 -1.000000E+10  0.000000E+00  0.000000E+00  0.000000E+00  0.000000E+00  0.000000E+00  0.000000E+00  0.000000E+00  0.000000E+00  0.000000E+00  0.000000E+00  0.000000E+00  0.000000E+00       Funtag_D
      70  2.118679E+05  1.787502E+05       33117.7       17.4430      0.206002       1.38835      0.219700      0.233015    -0.0475250    -0.0589446    0.00521753      0.877523       Opttag_D
      70       57.7408       3.10702       1.00000  0.000000E+00       1.00000  0.000000E+00      0.881085       55.9374       1.00000       20.1060  0.000000E+00       6.14354  0.000000E+00       6.14354  0.000000E+00  0.000000E+00      -12.2871       57.8987       1.83292        1       Scatag_D
      70       765.331       4198.30  0.000000E+00  0.000000E+00       2403.74  0.000000E+00       1683.34       1518.23  0.000000E+00       7789.85       34.7433       14724.1 -1.000000E+10 -1.000000E+10 -1.000000E+10 -1.000000E+10 -1.000000E+10  0.000000E+00  0.000000E+00  0.000000E+00  0.000000E+00  0.000000E+00  0.000000E+00  0.000000E+00  0.000000E+00  0.000000E+00  0.000000E+00  0.000000E+00  0.000000E+00       Funtag_D
      71  2.117854E+05  1.787812E+05       33004.2       41.7347      0.208718       1.41478      0.219767      0.233381    -0.0477561    -0.0599619    0.00522303      0.875033       Opttag_D
      71       57.7981       3.12101       1.00000  0.000000E+00       1.00000  0.000000E+00      0.881238       55.9352       1.00000       20.1124  0.000000E+00       6.13860  0.000000E+00       6.13860  0.000000E+00  0.000000E+00      -12.2772       57.8987       1.83292        1       Scatag_D
      71       766.944       4174.51  0.000000E+00  0.000000E+00       2392.51  0.000000E+00       1671.74       1615.51  0.000000E+00       7722.21       33.3479       14627.4 -1.000000E+10 -1.000000E+10 -1.000000E+10 -1.000000E+10 -1.000000E+10  0.000000E+00  0.000000E+00  0.000000E+00  0.000000E+00  0.000000E+00  0.000000E+00  0.000000E+00  0.000000E+00  0.000000E+00  0.000000E+00  0.000000E+00  0.000000E+00       Funtag_D
      72  2.116255E+05  1.787994E+05       32826.1       16.6639      0.209665       1.42418      0.219809      0.233506    -0.0478916    -0.0603243    0.00519407      0.871961       Opttag_D
      72       57.8153       3.12632       1.00000  0.000000E+00       1.00000  0.000000E+00      0.881293       55.9302       1.00000       20.1171  0.000000E+00       6.13761  0.000000E+00       6.13761  0.000000E+00  0.000000E+00      -12.2752       57.8987       1.83292        1       Scatag_D
      72       758.463       4145.24  0.000000E+00  0.000000E+00       2390.02  0.000000E+00       1668.53       1521.91  0.000000E+00       7702.50       33.1976       14606.3 -1.000000E+10 -1.000000E+10 -1.000000E+10 -1.000000E+10 -1.000000E+10  0.000000E+00  0.000000E+00  0.000000E+00  0.000000E+00  0.000000E+00  0.000000E+00  0.000000E+00  0.000000E+00  0.000000E+00  0.000000E+00  0.000000E+00  0.000000E+00       Funtag_D
      73  2.115464E+05  1.788230E+05       32723.4       12.3636      0.210861       1.43577      0.219866      0.233667    -0.0480678    -0.0607720    0.00518170      0.870327       Opttag_D
      73       57.8363       3.13293       1.00000  0.000000E+00       1.00000  0.000000E+00      0.881364       55.9215       1.00000       20.1250  0.000000E+00       6.13670  0.000000E+00       6.13670  0.000000E+00  0.000000E+00      -12.2734       57.8987       1.83292        1       Scatag_D
      73       754.855       4129.72  0.000000E+00  0.000000E+00       2387.44  0.000000E+00       1664.48       1501.37  0.000000E+00       7678.60       33.1866       14573.8 -1.000000E+10 -1.000000E+10 -1.000000E+10 -1.000000E+10 -1.000000E+10  0.000000E+00  0.000000E+00  0.000000E+00  0.000000E+00  0.000000E+00  0.000000E+00  0.000000E+00  0.000000E+00  0.000000E+00  0.000000E+00  0.000000E+00  0.000000E+00       Funtag_D
      74  2.114637E+05  1.788557E+05       32608.0       14.4225      0.212647       1.45173      0.219946      0.233905    -0.0483123    -0.0614432    0.00517260      0.868329       Opttag_D
      74       57.8689       3.14278       1.00000  0.000000E+00       1.00000  0.000000E+00      0.881465       55.9134       1.00000       20.1355  0.000000E+00       6.13482  0.000000E+00       6.13482  0.000000E+00  0.000000E+00      -12.2696       57.8987       1.83292        1       Scatag_D
      74       752.205       4110.79  0.000000E+00  0.000000E+00       2383.18  0.000000E+00       1658.61       1499.65  0.000000E+00       7643.54       33.3711       14526.6 -1.000000E+10 -1.000000E+10 -1.000000E+10 -1.000000E+10 -1.000000E+10  0.000000E+00  0.000000E+00  0.000000E+00  0.000000E+00  0.000000E+00  0.000000E+00  0.000000E+00  0.000000E+00  0.000000E+00  0.000000E+00  0.000000E+00  0.000000E+00       Funtag_D
      75  2.113483E+05  1.789081E+05       32440.2       26.7289      0.215662       1.47500      0.220071      0.234294    -0.0487026    -0.0625718    0.00512541      0.865771       Opttag_D
      75       57.9250       3.15940       1.00000  0.000000E+00       1.00000  0.000000E+00      0.881619       55.9057       1.00000       20.1527  0.000000E+00       6.13025  0.000000E+00       6.13025  0.000000E+00  0.000000E+00      -12.2605       57.8987       1.83292        1       Scatag_D
      75       738.544       4086.60  0.000000E+00  0.000000E+00       2376.04  0.000000E+00       1647.54       1531.96  0.000000E+00       7585.34       33.6663       14440.5 -1.000000E+10 -1.000000E+10 -1.000000E+10 -1.000000E+10 -1.000000E+10  0.000000E+00  0.000000E+00  0.000000E+00  0.000000E+00  0.000000E+00  0.000000E+00  0.000000E+00  0.000000E+00  0.000000E+00  0.000000E+00  0.000000E+00  0.000000E+00       Funtag_D
      76  2.112333E+05  1.789646E+05       32268.7       15.4719      0.218748       1.49762      0.220210      0.234686    -0.0491243    -0.0637397    0.00510481      0.862962       Opttag_D
      76       57.9826       3.17694       1.00000  0.000000E+00       1.00000  0.000000E+00      0.881771       55.9033       1.00000       20.1740  0.000000E+00       6.12523  0.000000E+00       6.12523  0.000000E+00  0.000000E+00      -12.2505       57.8987       1.83292        1       Scatag_D
      76       732.619       4060.12  0.000000E+00  0.000000E+00       2369.53  0.000000E+00       1638.87       1513.72  0.000000E+00       7534.17       33.7064       14385.9 -1.000000E+10 -1.000000E+10 -1.000000E+10 -1.000000E+10 -1.000000E+10  0.000000E+00  0.000000E+00  0.000000E+00  0.000000E+00  0.000000E+00  0.000000E+00  0.000000E+00  0.000000E+00  0.000000E+00  0.000000E+00  0.000000E+00  0.000000E+00       Funtag_D

 An atom has moved more than     1.50 A from the initial/last
 position - if this was an accepted trial point, probably need to update the
 non-bonded contact list.

      77  2.111570E+05  1.789649E+05       32192.1       11.7052      0.219148       1.50176      0.220207      0.234722    -0.0491268    -0.0638682    0.00507859      0.861765       Opttag_D
      77       57.9924       3.17873       1.00000  0.000000E+00       1.00000  0.000000E+00      0.881780       55.9074       1.00000       20.1761  0.000000E+00       6.12317  0.000000E+00       6.12317  0.000000E+00  0.000000E+00      -12.2463       57.8987       1.83292        1       Scatag_D
      77       725.113       4048.87  0.000000E+00  0.000000E+00       2367.87  0.000000E+00       1636.32       1502.60  0.000000E+00       7523.35       33.3708       14354.6 -1.000000E+10 -1.000000E+10 -1.000000E+10 -1.000000E+10 -1.000000E+10  0.000000E+00  0.000000E+00  0.000000E+00  0.000000E+00  0.000000E+00  0.000000E+00  0.000000E+00  0.000000E+00  0.000000E+00  0.000000E+00  0.000000E+00  0.000000E+00       Funtag_D

 An atom has moved more than     1.50 A from the initial/last
 position - need to update the non-bonded contact list.


 ############################################################################################################
 ###################################################### setting up contact terms
 ############################################################################################################

      78  2.110411E+05  1.789685E+05       32072.5       16.9200      0.220281       1.51327      0.220211      0.234829    -0.0491537    -0.0642318    0.00505851      0.859625       Opttag_D
      78       58.0198       3.18404       1.00000  0.000000E+00       1.00000  0.000000E+00      0.881809       55.9170       1.00000       20.1846  0.000000E+00       6.11767  0.000000E+00       6.11767  0.000000E+00  0.000000E+00      -12.2353       57.8987       1.83292        1       Scatag_D
      78       719.390       4028.78  0.000000E+00  0.000000E+00       2363.52  0.000000E+00       1631.19       1516.09  0.000000E+00       7495.31       32.4389       14285.8 -1.000000E+10 -1.000000E+10 -1.000000E+10 -1.000000E+10 -1.000000E+10  0.000000E+00  0.000000E+00  0.000000E+00  0.000000E+00  0.000000E+00  0.000000E+00  0.000000E+00  0.000000E+00  0.000000E+00  0.000000E+00  0.000000E+00  0.000000E+00       Funtag_D
      79  2.109796E+05  1.789978E+05       31981.8       28.3378      0.222647       1.53767      0.220275      0.235083    -0.0493718    -0.0649958    0.00505836      0.857814       Opttag_D
      79       58.0699       3.19659       1.00000  0.000000E+00       1.00000  0.000000E+00      0.881897       55.9243       1.00000       20.2080  0.000000E+00       6.11022  0.000000E+00       6.11022  0.000000E+00  0.000000E+00      -12.2204       57.8987       1.83292        1       Scatag_D
      79       719.345       4011.83  0.000000E+00  0.000000E+00       2357.07  0.000000E+00       1625.29       1560.99  0.000000E+00       7453.36       31.5929       14222.3 -1.000000E+10 -1.000000E+10 -1.000000E+10 -1.000000E+10 -1.000000E+10  0.000000E+00  0.000000E+00  0.000000E+00  0.000000E+00  0.000000E+00  0.000000E+00  0.000000E+00  0.000000E+00  0.000000E+00  0.000000E+00  0.000000E+00  0.000000E+00       Funtag_D
      80  2.108850E+05  1.789982E+05       31886.8       13.9638      0.222847       1.53968      0.220277      0.235099    -0.0493748    -0.0650325    0.00503882      0.856378       Opttag_D
      80       58.0748       3.19746       1.00000  0.000000E+00       1.00000  0.000000E+00      0.881902       55.9247       1.00000       20.2111  0.000000E+00       6.10953  0.000000E+00       6.10953  0.000000E+00  0.000000E+00      -12.2191       57.8987       1.83292        1       Scatag_D
      80       713.800       3998.41  0.000000E+00  0.000000E+00       2356.17  0.000000E+00       1624.11       1511.65  0.000000E+00       7447.69       31.5327       14203.4 -1.000000E+10 -1.000000E+10 -1.000000E+10 -1.000000E+10 -1.000000E+10  0.000000E+00  0.000000E+00  0.000000E+00  0.000000E+00  0.000000E+00  0.000000E+00  0.000000E+00  0.000000E+00  0.000000E+00  0.000000E+00  0.000000E+00  0.000000E+00       Funtag_D
      81  2.107954E+05  1.790117E+05       31783.8       12.0726      0.223854       1.55014      0.220307      0.235205    -0.0494756    -0.0653187    0.00502914      0.854496       Opttag_D
      81       58.0958       3.20277       1.00000  0.000000E+00       1.00000  0.000000E+00      0.881942       55.9238       1.00000       20.2240  0.000000E+00       6.10734  0.000000E+00       6.10734  0.000000E+00  0.000000E+00      -12.2147       57.8987       1.83292        1       Scatag_D
      81       711.060       3980.85  0.000000E+00  0.000000E+00       2353.37  0.000000E+00       1620.25       1497.49  0.000000E+00       7428.72       31.5425       14160.5 -1.000000E+10 -1.000000E+10 -1.000000E+10 -1.000000E+10 -1.000000E+10  0.000000E+00  0.000000E+00  0.000000E+00  0.000000E+00  0.000000E+00  0.000000E+00  0.000000E+00  0.000000E+00  0.000000E+00  0.000000E+00  0.000000E+00  0.000000E+00       Funtag_D
      82  2.107302E+05  1.790305E+05       31699.7       13.3037      0.225109       1.56318      0.220353      0.235340    -0.0496158    -0.0656873    0.00502700      0.853194       Opttag_D
      82       58.1214       3.20962       1.00000  0.000000E+00       1.00000  0.000000E+00      0.881990       55.9234       1.00000       20.2386  0.000000E+00       6.10475  0.000000E+00       6.10475  0.000000E+00  0.000000E+00      -12.2095       57.8987       1.83292        1       Scatag_D
      82       710.456       3968.73  0.000000E+00  0.000000E+00       2350.06  0.000000E+00       1615.48       1498.39  0.000000E+00       7407.08       31.6789       14117.8 -1.000000E+10 -1.000000E+10 -1.000000E+10 -1.000000E+10 -1.000000E+10  0.000000E+00  0.000000E+00  0.000000E+00  0.000000E+00  0.000000E+00  0.000000E+00  0.000000E+00  0.000000E+00  0.000000E+00  0.000000E+00  0.000000E+00  0.000000E+00       Funtag_D
      83  2.106284E+05  1.790788E+05       31549.6       29.1388      0.228185       1.59504      0.220460      0.235667    -0.0499759    -0.0665856    0.00500029      0.852174       Opttag_D
      83       58.1845       3.22682       1.00000  0.000000E+00       1.00000  0.000000E+00      0.882096       55.9201       1.00000       20.2745  0.000000E+00       6.09806  0.000000E+00       6.09806  0.000000E+00  0.000000E+00      -12.1961       57.8987       1.83292        1       Scatag_D
      83       702.925       3959.24  0.000000E+00  0.000000E+00       2341.70  0.000000E+00       1603.29       1541.53  0.000000E+00       7356.24       32.0674       14012.6 -1.000000E+10 -1.000000E+10 -1.000000E+10 -1.000000E+10 -1.000000E+10  0.000000E+00  0.000000E+00  0.000000E+00  0.000000E+00  0.000000E+00  0.000000E+00  0.000000E+00  0.000000E+00  0.000000E+00  0.000000E+00  0.000000E+00  0.000000E+00       Funtag_D
      84  2.105031E+05  1.791221E+05       31381.1       16.0148      0.230679       1.62028      0.220555      0.235941    -0.0502989    -0.0673114    0.00498870      0.851025       Opttag_D
      84       58.2351       3.24132       1.00000  0.000000E+00       1.00000  0.000000E+00      0.882179       55.9197       1.00000       20.3052  0.000000E+00       6.09262  0.000000E+00       6.09262  0.000000E+00  0.000000E+00      -12.1852       57.8987       1.83292        1       Scatag_D
      84       699.669       3948.58  0.000000E+00  0.000000E+00       2335.57  0.000000E+00       1596.14       1505.57  0.000000E+00       7322.07       32.2795       13941.2 -1.000000E+10 -1.000000E+10 -1.000000E+10 -1.000000E+10 -1.000000E+10  0.000000E+00  0.000000E+00  0.000000E+00  0.000000E+00  0.000000E+00  0.000000E+00  0.000000E+00  0.000000E+00  0.000000E+00  0.000000E+00  0.000000E+00  0.000000E+00       Funtag_D
      85  2.104123E+05  1.791361E+05       31276.2       12.9743      0.231545       1.62934      0.220585      0.236033    -0.0504033    -0.0675573    0.00495876      0.850619       Opttag_D
      85       58.2542       3.24650       1.00000  0.000000E+00       1.00000  0.000000E+00      0.882200       55.9180       1.00000       20.3158  0.000000E+00       6.09012  0.000000E+00       6.09012  0.000000E+00  0.000000E+00      -12.1802       57.8987       1.83292        1       Scatag_D
      85       691.299       3944.81  0.000000E+00  0.000000E+00       2332.66  0.000000E+00       1592.71       1487.45  0.000000E+00       7307.54       32.1570       13887.6 -1.000000E+10 -1.000000E+10 -1.000000E+10 -1.000000E+10 -1.000000E+10  0.000000E+00  0.000000E+00  0.000000E+00  0.000000E+00  0.000000E+00  0.000000E+00  0.000000E+00  0.000000E+00  0.000000E+00  0.000000E+00  0.000000E+00  0.000000E+00       Funtag_D
      86  2.102724E+05  1.791783E+05       31094.1       17.9730      0.233991       1.65419      0.220681      0.236306    -0.0507183    -0.0682377    0.00496688      0.850239       Opttag_D
      86       58.3080       3.26139       1.00000  0.000000E+00       1.00000  0.000000E+00      0.882263       55.9127       1.00000       20.3510  0.000000E+00       6.08298  0.000000E+00       6.08298  0.000000E+00  0.000000E+00      -12.1660       57.8987       1.83292        1       Scatag_D
      86       693.564       3941.28  0.000000E+00  0.000000E+00       2324.86  0.000000E+00       1585.98       1492.12  0.000000E+00       7272.68       31.6731       13752.0 -1.000000E+10 -1.000000E+10 -1.000000E+10 -1.000000E+10 -1.000000E+10  0.000000E+00  0.000000E+00  0.000000E+00  0.000000E+00  0.000000E+00  0.000000E+00  0.000000E+00  0.000000E+00  0.000000E+00  0.000000E+00  0.000000E+00  0.000000E+00       Funtag_D
      87  2.102488E+05  1.792298E+05       31019.0       36.1945      0.236821       1.68193      0.220800      0.236630    -0.0511029    -0.0690609    0.00500061      0.850251       Opttag_D
      87       58.3698       3.27884       1.00000  0.000000E+00       1.00000  0.000000E+00      0.882343       55.9028       1.00000       20.4000  0.000000E+00       6.07504  0.000000E+00       6.07504  0.000000E+00  0.000000E+00      -12.1501       57.8987       1.83292        1       Scatag_D
      87       703.014       3941.40  0.000000E+00  0.000000E+00       2316.30  0.000000E+00       1579.12       1566.06  0.000000E+00       7239.23       31.0125       13642.8 -1.000000E+10 -1.000000E+10 -1.000000E+10 -1.000000E+10 -1.000000E+10  0.000000E+00  0.000000E+00  0.000000E+00  0.000000E+00  0.000000E+00  0.000000E+00  0.000000E+00  0.000000E+00  0.000000E+00  0.000000E+00  0.000000E+00  0.000000E+00       Funtag_D
      88  2.101065E+05  1.791954E+05       30911.1       15.4278      0.235060       1.66522      0.220720      0.236430    -0.0508461    -0.0685429    0.00496377      0.848082       Opttag_D
      88       58.3364       3.26838       1.00000  0.000000E+00       1.00000  0.000000E+00      0.882285       55.9027       1.00000       20.3742  0.000000E+00       6.07866  0.000000E+00       6.07866  0.000000E+00  0.000000E+00      -12.1573       57.8987       1.83292        1       Scatag_D
      88       692.695       3921.31  0.000000E+00  0.000000E+00       2319.15  0.000000E+00       1578.88       1480.65  0.000000E+00       7250.09       30.8315       13637.5 -1.000000E+10 -1.000000E+10 -1.000000E+10 -1.000000E+10 -1.000000E+10  0.000000E+00  0.000000E+00  0.000000E+00  0.000000E+00  0.000000E+00  0.000000E+00  0.000000E+00  0.000000E+00  0.000000E+00  0.000000E+00  0.000000E+00  0.000000E+00       Funtag_D
      89  2.100315E+05  1.792029E+05       30828.6       10.7455      0.235420       1.66878      0.220738      0.236482    -0.0509023    -0.0686447    0.00494866      0.847085       Opttag_D
      89       58.3453       3.27074       1.00000  0.000000E+00       1.00000  0.000000E+00      0.882297       55.8977       1.00000       20.3837  0.000000E+00       6.07788  0.000000E+00       6.07788  0.000000E+00  0.000000E+00      -12.1558       57.8987       1.83292        1       Scatag_D
      89       688.484       3912.10  0.000000E+00  0.000000E+00       2317.29  0.000000E+00       1575.10       1461.63  0.000000E+00       7242.89       30.7227       13600.4 -1.000000E+10 -1.000000E+10 -1.000000E+10 -1.000000E+10 -1.000000E+10  0.000000E+00  0.000000E+00  0.000000E+00  0.000000E+00  0.000000E+00  0.000000E+00  0.000000E+00  0.000000E+00  0.000000E+00  0.000000E+00  0.000000E+00  0.000000E+00       Funtag_D
      90  2.099493E+05  1.792226E+05       30726.7       13.3284      0.236345       1.67779      0.220790      0.236609    -0.0510490    -0.0689269    0.00496259      0.846069       Opttag_D
      90       58.3666       3.27667       1.00000  0.000000E+00       1.00000  0.000000E+00      0.882330       55.8873       1.00000       20.4052  0.000000E+00       6.07629  0.000000E+00       6.07629  0.000000E+00  0.000000E+00      -12.1526       57.8987       1.83292        1       Scatag_D
      90       692.365       3902.72  0.000000E+00  0.000000E+00       2313.43  0.000000E+00       1567.32       1454.03  0.000000E+00       7228.28       30.7249       13537.8 -1.000000E+10 -1.000000E+10 -1.000000E+10 -1.000000E+10 -1.000000E+10  0.000000E+00  0.000000E+00  0.000000E+00  0.000000E+00  0.000000E+00  0.000000E+00  0.000000E+00  0.000000E+00  0.000000E+00  0.000000E+00  0.000000E+00  0.000000E+00       Funtag_D
      91  2.098589E+05  1.792459E+05       30613.0       20.8073      0.237359       1.68814      0.220852      0.236753    -0.0512226    -0.0692706    0.00494947      0.845703       Opttag_D
      91       58.3906       3.28336       1.00000  0.000000E+00       1.00000  0.000000E+00      0.882362       55.8722       1.00000       20.4286  0.000000E+00       6.07534  0.000000E+00       6.07534  0.000000E+00  0.000000E+00      -12.1507       57.8987       1.83292        1       Scatag_D
      91       688.710       3899.34  0.000000E+00  0.000000E+00       2308.51  0.000000E+00       1555.31       1458.47  0.000000E+00       7210.25       30.9250       13461.5 -1.000000E+10 -1.000000E+10 -1.000000E+10 -1.000000E+10 -1.000000E+10  0.000000E+00  0.000000E+00  0.000000E+00  0.000000E+00  0.000000E+00  0.000000E+00  0.000000E+00  0.000000E+00  0.000000E+00  0.000000E+00  0.000000E+00  0.000000E+00       Funtag_D
      92  2.097534E+05  1.792849E+05       30468.5       14.2097      0.239114       1.70591      0.220953      0.236980    -0.0515138    -0.0698421    0.00495113      0.845850       Opttag_D
      92       58.4305       3.29470       1.00000  0.000000E+00       1.00000  0.000000E+00      0.882418       55.8494       1.00000       20.4671  0.000000E+00       6.07334  0.000000E+00       6.07334  0.000000E+00  0.000000E+00      -12.1467       57.8987       1.83292        1       Scatag_D
      92       689.172       3900.70  0.000000E+00  0.000000E+00       2301.22  0.000000E+00       1541.32       1426.26  0.000000E+00       7185.12       31.2249       13393.5 -1.000000E+10 -1.000000E+10 -1.000000E+10 -1.000000E+10 -1.000000E+10  0.000000E+00  0.000000E+00  0.000000E+00  0.000000E+00  0.000000E+00  0.000000E+00  0.000000E+00  0.000000E+00  0.000000E+00  0.000000E+00  0.000000E+00  0.000000E+00       Funtag_D
      93  2.096732E+05  1.792815E+05       30391.6       12.9553      0.238949       1.70615      0.220946      0.236967    -0.0514888    -0.0698390    0.00490130      0.845288       Opttag_D
      93       58.4310       3.29404       1.00000  0.000000E+00       1.00000  0.000000E+00      0.882408       55.8406       1.00000       20.4665  0.000000E+00       6.07422  0.000000E+00       6.07422  0.000000E+00  0.000000E+00      -12.1484       57.8987       1.83292        1       Scatag_D
      93       675.368       3895.52  0.000000E+00  0.000000E+00       2299.62  0.000000E+00       1537.74       1410.66  0.000000E+00       7180.04       31.3744       13361.3 -1.000000E+10 -1.000000E+10 -1.000000E+10 -1.000000E+10 -1.000000E+10  0.000000E+00  0.000000E+00  0.000000E+00  0.000000E+00  0.000000E+00  0.000000E+00  0.000000E+00  0.000000E+00  0.000000E+00  0.000000E+00  0.000000E+00  0.000000E+00       Funtag_D
      94  2.096118E+05  1.792724E+05       30339.4       11.8435      0.238567       1.70507      0.220923      0.236930    -0.0514204    -0.0697721    0.00489426      0.844606       Opttag_D
      94       58.4277       3.29195       1.00000  0.000000E+00       1.00000  0.000000E+00      0.882391       55.8330       1.00000       20.4611  0.000000E+00       6.07534  0.000000E+00       6.07534  0.000000E+00  0.000000E+00      -12.1507       57.8987       1.83292        1       Scatag_D
      94       673.431       3889.24  0.000000E+00  0.000000E+00       2298.61  0.000000E+00       1536.12       1398.95  0.000000E+00       7178.12       31.4687       13333.5 -1.000000E+10 -1.000000E+10 -1.000000E+10 -1.000000E+10 -1.000000E+10  0.000000E+00  0.000000E+00  0.000000E+00  0.000000E+00  0.000000E+00  0.000000E+00  0.000000E+00  0.000000E+00  0.000000E+00  0.000000E+00  0.000000E+00  0.000000E+00       Funtag_D
      95  2.095657E+05  1.792686E+05       30297.2       10.7510      0.238555       1.70714      0.220913      0.236922    -0.0513919    -0.0697706    0.00488228      0.843806       Opttag_D
      95       58.4309       3.29167       1.00000  0.000000E+00       1.00000  0.000000E+00      0.882389       55.8256       1.00000       20.4635  0.000000E+00       6.07523  0.000000E+00       6.07523  0.000000E+00  0.000000E+00      -12.1505       57.8987       1.83292        1       Scatag_D
      95       670.138       3881.87  0.000000E+00  0.000000E+00       2297.28  0.000000E+00       1534.93       1392.17  0.000000E+00       7175.57       31.3578       13313.8 -1.000000E+10 -1.000000E+10 -1.000000E+10 -1.000000E+10 -1.000000E+10  0.000000E+00  0.000000E+00  0.000000E+00  0.000000E+00  0.000000E+00  0.000000E+00  0.000000E+00  0.000000E+00  0.000000E+00  0.000000E+00  0.000000E+00  0.000000E+00       Funtag_D
      96  2.095077E+05  1.792644E+05       30243.4       9.05431      0.238687       1.71126      0.220900      0.236921    -0.0513606    -0.0697977    0.00487341      0.842453       Opttag_D
      96       58.4376       3.29185       1.00000  0.000000E+00       1.00000  0.000000E+00      0.882391       55.8180       1.00000       20.4693  0.000000E+00       6.07437  0.000000E+00       6.07437  0.000000E+00  0.000000E+00      -12.1487       57.8987       1.83292        1       Scatag_D
      96       667.704       3869.43  0.000000E+00  0.000000E+00       2295.60  0.000000E+00       1533.38       1382.40  0.000000E+00       7172.77       31.0807       13291.0 -1.000000E+10 -1.000000E+10 -1.000000E+10 -1.000000E+10 -1.000000E+10  0.000000E+00  0.000000E+00  0.000000E+00  0.000000E+00  0.000000E+00  0.000000E+00  0.000000E+00  0.000000E+00  0.000000E+00  0.000000E+00  0.000000E+00  0.000000E+00       Funtag_D
      97  2.094801E+05  1.792633E+05       30216.8       22.1539      0.239313       1.72194      0.220894      0.236967    -0.0513526    -0.0699599    0.00488773      0.841080       Opttag_D
      97       58.4572       3.29428       1.00000  0.000000E+00       1.00000  0.000000E+00      0.882409       55.8048       1.00000       20.4869  0.000000E+00       6.07170  0.000000E+00       6.07170  0.000000E+00  0.000000E+00      -12.1434       57.8987       1.83292        1       Scatag_D
      97       671.634       3856.83  0.000000E+00  0.000000E+00       2291.89  0.000000E+00       1529.27       1403.54  0.000000E+00       7166.00       30.6874       13267.0 -1.000000E+10 -1.000000E+10 -1.000000E+10 -1.000000E+10 -1.000000E+10  0.000000E+00  0.000000E+00  0.000000E+00  0.000000E+00  0.000000E+00  0.000000E+00  0.000000E+00  0.000000E+00  0.000000E+00  0.000000E+00  0.000000E+00  0.000000E+00       Funtag_D
      98  2.094288E+05  1.792621E+05       30166.6       8.20275      0.239406       1.72305      0.220890      0.236976    -0.0513440    -0.0699752    0.00487345      0.840525       Opttag_D
      98       58.4595       3.29439       1.00000  0.000000E+00       1.00000  0.000000E+00      0.882412       55.8040       1.00000       20.4892  0.000000E+00       6.07125  0.000000E+00       6.07125  0.000000E+00  0.000000E+00      -12.1425       57.8987       1.83292        1       Scatag_D
      98       667.717       3851.75  0.000000E+00  0.000000E+00       2291.51  0.000000E+00       1528.54       1373.87  0.000000E+00       7164.87       30.6839       13257.7 -1.000000E+10 -1.000000E+10 -1.000000E+10 -1.000000E+10 -1.000000E+10  0.000000E+00  0.000000E+00  0.000000E+00  0.000000E+00  0.000000E+00  0.000000E+00  0.000000E+00  0.000000E+00  0.000000E+00  0.000000E+00  0.000000E+00  0.000000E+00       Funtag_D
      99  2.094091E+05  1.792618E+05       30147.3       7.34452      0.239483       1.72375      0.220889      0.236988    -0.0513418    -0.0699939    0.00485541      0.840356       Opttag_D
      99       58.4614       3.29461       1.00000  0.000000E+00       1.00000  0.000000E+00      0.882414       55.8043       1.00000       20.4906  0.000000E+00       6.07090  0.000000E+00       6.07090  0.000000E+00  0.000000E+00      -12.1418       57.8987       1.83292        1       Scatag_D
      99       662.781       3850.19  0.000000E+00  0.000000E+00       2291.18  0.000000E+00       1527.66       1373.29  0.000000E+00       7163.51       30.7505       13247.9 -1.000000E+10 -1.000000E+10 -1.000000E+10 -1.000000E+10 -1.000000E+10  0.000000E+00  0.000000E+00  0.000000E+00  0.000000E+00  0.000000E+00  0.000000E+00  0.000000E+00  0.000000E+00  0.000000E+00  0.000000E+00  0.000000E+00  0.000000E+00       Funtag_D

   MaxLik Scaling Cycle 0104

   Wilson plot

   Statistics in resolution bins after refinement cycle 0003
```


|  |  |
| --- | --- |
|  | - Rxpct factors plot- Rxpct factors table- <Log-Lik Gain>- Ln Sigma A- Correlation Coefficients |


```
   Iters     Tot_Funct    Xray_Funct    Geom_Funct          Grms     rmsD2init     maxD2init             R         Rfree           LLG       LLGfree     rms(bond)    rms(angle)
   Iters             K   B_IMPF_FRAG        K_MISS   B_TEMP_MISS   K_IMPF_MISS   B_IMPF_MISS        K_SOLV   B_TEMP_SOLV   K_IMPF_SOLV   B_IMPF_SOLV        B_TEMP     B_TEMP_11     B_TEMP_12     B_TEMP_22     B_TEMP_13     B_TEMP_23     B_TEMP_33      K_WILSON      B_WILSON      FGCYCLES
   Iters          BOND         ANGLE      IMPROPER       TORSION    SINTORSION      TRIGONAL        PLANAR  BCORRELATION       CONTACT     IDEALDIST        CHIRAL    SIMILARITY       OCCSSUM  UTILDISTANCE     UTILANGLE   UTILTORSION        RHOFIT        ADP-01        ADP-02        ADP-03        ADP-04        ADP-05        ADP-06        ADP-07        ADP-08        ADP-09        ADP-10      RHOFITPC            QM
     100  2.093648E+05  1.792597E+05       30105.1       9.54651      0.239893       1.72938      0.220881      0.237030    -0.0513260    -0.0700921    0.00483711      0.839831       Opttag_D
     100       58.4738       3.29595       1.00000  0.000000E+00       1.00000  0.000000E+00      0.882428       55.8000       1.00000       20.5004  0.000000E+00       6.06915  0.000000E+00       6.06915  0.000000E+00  0.000000E+00      -12.1383       57.8987       1.83292        1       Scatag_D
     100       657.796       3845.38  0.000000E+00  0.000000E+00       2288.76  0.000000E+00       1525.12       1376.40  0.000000E+00       7157.60       30.8750       13223.2 -1.000000E+10 -1.000000E+10 -1.000000E+10 -1.000000E+10 -1.000000E+10  0.000000E+00  0.000000E+00  0.000000E+00  0.000000E+00  0.000000E+00  0.000000E+00  0.000000E+00  0.000000E+00  0.000000E+00  0.000000E+00  0.000000E+00  0.000000E+00       Funtag_D
   Geometry Restraint Deviations SCREEN Information

 
 No -bcorrel_zeroocchydrogens_factor flag found - keeping default: will set B for OCC=0 hydrogens to    1.200 times heavy-atom B


 TLS information. Total number of groups:     2


 Group   Natoms    FixRB   FixTLS   Group Tag    Group Spec
     1     2770      Y        Y     tls1    { A|* }
     2     2774      Y        Y     tls2    { B|* }
 
NOTE BUSTER_TLS_SET tls1 { A|* }
NOTE BUSTER_TLS_T   tls1     -1.40902E-01 -6.30068E-02  2.40854E-01 -1.40248E-02  2.99908E-02 -4.86377E-03
NOTE BUSTER_TLS_L   tls1      4.94961E-01  2.74622E-01  7.99820E-02  2.76942E-01 -2.11335E-01  5.62449E-04
NOTE BUSTER_TLS_S   tls1      2.26571E-01 -1.51776E-01  8.73531E-02 -1.03155E-01 -2.74707E-02 -1.84204E-02 -5.17057E-02  1.25425E-02
NOTE BUSTER_TLS_O   tls1     -2.33748E+01 -8.36624E+01  1.14308E+01
NOTE BUSTER_TLS_FIX tls1 ALL
 
NOTE BUSTER_TLS_SET tls2 { B|* }
NOTE BUSTER_TLS_T   tls2     -1.42296E-01 -1.01915E-01  3.03964E-01  1.65150E-02  4.29271E-02  1.39210E-02
NOTE BUSTER_TLS_L   tls2      5.71646E-01  2.05044E-01  3.60703E-01  3.24162E-01 -3.15261E-01 -1.73112E-01
NOTE BUSTER_TLS_S   tls2     -1.36019E-02  5.36055E-02 -3.99431E-02  1.31338E-02  1.12817E-01 -1.79603E-02 -2.03873E-02  1.93526E-02
NOTE BUSTER_TLS_O   tls2      2.20570E+01 -7.19562E+01  3.49479E+00
NOTE BUSTER_TLS_FIX tls2 ALL

 For final position, Geometry function summary
     term                           number     weight       rms        GooF      function     fn/numb
     ================================================================================================
     BOND bond lengths (angs)       5660       2.000      0.0048       0.241       657.80       0.116
     ANGL bond angles (degs)        7681       2.000        0.84       0.500      3845.38       0.501
     TORS split-harmonic tors       1270       0.000       17.19       1.081         0.00       0.000
     SINTOR sinusoidal tors         1270       2.000       17.19                  2288.76       1.802
     PLAN general plane (angs)       964       5.000      0.0113       0.563      1525.12       1.582
     BCORrelations (angs^2)         5660      10.000       0.437       0.156      1376.40       0.243
     CONT (bad contacts)               0       5.000                                 0.00
     IDEAL(ideal-dist contact)      4879       4.000                              7157.60
     CHIRAL (gelly semiharmon)       740       5.000                                30.87
     SIM similarity restraint for NCS and/or TARGET                              13223.17
     OCCSUM restraint on sum of occupanies                                           0.00       as none defined
     DISTAN utility restraints interatomic distance                                  0.00       as none defined
     UTILANGLE utility angle restraints                                              0.00       as none defined
     UTILTOR utility restraints on torsion angles                                    0.00       as none defined
     Peptide omega torsion angles                           2.39 degs - monitor only
 
 WARNING: Have |delta/sigma| deviations > 5.0 sigma. Number of outliers for each term: 
 WARNING:         2 bond angles.  Worst is     5.3 sigs   135.02 degs B|28:C.A(ASN)=B|29:N(ASN)=B|29:CA(ASN)

 Weighted rms      0.0048         0.840  (temporary rms bond and angle for BUSTER to pick up!)
 Weighted intra-residual rms(bond) for StdProtein (excluding H)      0.0046
 Weighted intra-residual rms(bond) for Buffer     (excluding H)      0.0030
 Outputing geometry progress graph in plotmtv format to file:Geom_Res.mtv

 ############### Running final geometry/screen analysis (screen_final.txt)

 s/r gelly normal termination
```

Statistics and scale factors vs. cycle number
```

|  |  |  |
| --- | --- | --- |
|  | - Rxpct factors- <Log-Lik Gain>- GEOM Residuals- Ln Sigma_A_ | - Scale Factors- B Factors- Imperfection Factors |

```
   Final Geometry Information


---


### 2. Final results

Statistics and scale factors vs. cycle number
```

|  |  |  |
| --- | --- | --- |
|  | - Rxpct factors- <Log-Lik Gain>- GEOM Residuals- Ln Sigma_A_ | - Scale Factors- B Factors- Imperfection Factors |

```
   Average Figure of Merit
   Structure Factor Amplitudes


 # normal termination

  Program stopped.
```

closesok message: time spent in tree = 1589.64 (user) 37.32 (sys)

---

# Normal termination

```
 Resources usage summary:
     Time spent in client ...................  1589.65 (user)       37.34 (system)
     Time spent in scripts (    27 calls) ...     2.37 (user)        0.14 (system)
     Time spent in server ...................     0.00 (user)        0.00 (system)

     Max resident memory used (kb) .......... 444272
     Number of page faults .................. 0 (major)    855271 (minor)
     Number of context switches ............. 168269 (niced)    6216 (other)
```
